# Synthesis and photophysical properties of novel benzophospholo[3,2-*b*]indole derivatives

**DOI:** 10.3762/bjoc.13.226

**Published:** 2017-10-30

**Authors:** Mio Matsumura, Mizuki Yamada, Atsuya Muranaka, Misae Kanai, Naoki Kakusawa, Daisuke Hashizume, Masanobu Uchiyama, Shuji Yasuike

**Affiliations:** 1 School of Pharmaceutical Sciences, Aichi Gakuin University, 1-100 Kusumoto-cho, Chikusa-ku, Nagoya 464-8650, Japan; 2 Elements Chemistry Laboratory, RIKEN, and Advanced Elements Chemistry Research Team, RIKEN Center for Sustainable Resource Science (CSRS), Wako 351-0198, Japan; 3 Faculty of Pharmaceutical Sciences, Hokuriku University, Ho-3 Kanagawa-machi, Kanazawa 920-1181, Japan; 4 Materials Characterization Support Unit, RIKEN Center for Emergent Matter Science (CEMS), Wako 351-0198, Japan; 5 Graduate School of Pharmaceutical Sciences, The University of Tokyo, Tokyo 113-0033, Japan

**Keywords:** benzophospholo[3,2-*b*]indole, DFT calculation, molecular structure, phosphole derivatives, photophysical property

## Abstract

The parent benzophospholo[3,2-*b*]indole was prepared by the reaction of dichlorophenylphosphine with a dilithium intermediate, which was prepared in two steps from 2-ethynyl-*N*,*N*-dimethylaniline. Using the obtained benzophosphole-fused indole as a common starting material, simple modifications were carried out at the phosphorus center of the phosphole, synthesizing various functionalized analogs. The X-ray structure analysis of trivalent phosphole and phosphine oxide showed that the fused tetracyclic moieties are planar. The benzophosphole-fused indoles, such as phosphine oxide, phospholium salt, and borane complex, exhibited strong photoluminescence in dichloromethane (Φ = 67–75%).

## Introduction

**Figure 1:**
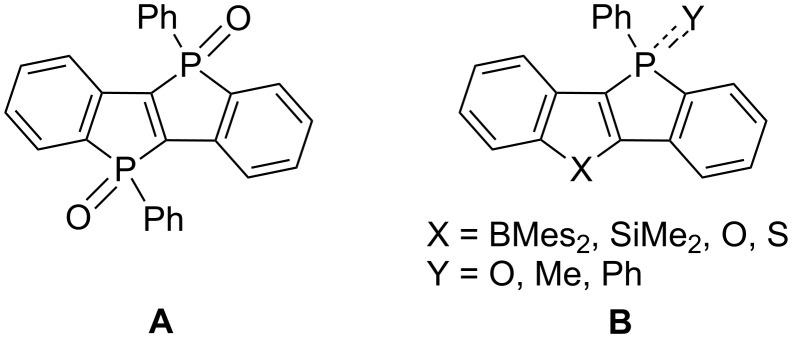
Phosphole-based tetracyclic heteroacenes.

The chemistry of phospholes, fully unsaturated five-membered heterocyclic rings containing a phosphorus element, has drawn much attention in terms of the development of synthetic methods and elucidation of its spectroscopic properties for applications in organic field-effect transistors (OFETs) and luminescent materials [1–9]. The phosphorous atom of trivalent phosphorus compounds has a high chemical reactivity. Therefore, several reactions on the phosphorus atom such as oxidation, alkylation, and coordination to a Lewis acid can produce the corresponding phosphole derivatives with different electronic properties [10–17]. Phosphole-based ladder-type π-conjugated heteroacenes were shown to exhibit a high charge mobility and/or fluorescence quantum yields [18–24]. For example, dibenzo-fused phospholo[3,2-*b*]phosphole dioxides (Figure 1A) [25–26] and benzophosphole-fused tetracyclic heteroacenes, containing boron (B) [27–29], silicon (Si) [30], oxygen (O) [31], and sulfur (S) [32–34] (Figure 1B), were synthesized and their physical properties were studied. However, to the best of our knowledge, the synthesis of benzophosphole-fused indole derivatives as tetracyclic heteroacenes has not been reported. In 2015, Lu et al. reported the synthesis of only one phosphole and indole-fused pentacyclic heteroacene [35]. Recently, we reported simple and efficient synthetic routes to benzothiophene-fused benzoheteroles containing the group 15 and 16 elements using the ring-closing reaction of dilithium compounds with electrophiles bearing heteroatoms [36]. In continuation of our research, we were interested in the synthesis, molecular structure, and physicochemical properties of the parent benzophosphole-fused indole derivative and its various functionalized analogs such as the corresponding phosphine oxide, phosphonium salt, and borane–phosphine complex.

## Results and Discussion

**Scheme 1:**
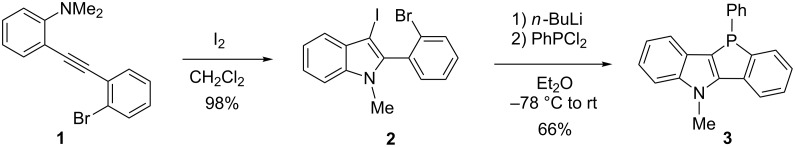
Synthesis of benzophospholo[3,2-*b*]indole **3**.

The synthesis of the parent tetracyclic molecule 10-phenyl-[1]benzophospholo[3,2-*b*]-*N*-methylindole (**3**), is shown in Scheme 1. The key precursor **2** was synthesized by I_2_-mediated electrophilic cyclization of 2-ethynyl-*N,N*-dimethylaniline **1** [36–37]. Treatment of compound **2** with *n*-butyllithium in anhydrous THF at −78 °C and subsequently with PhPCl_2_ resulted in ring closure, affording the desired benzophospholo[3,2-*b*]indole **3** in 66% yield.

**Scheme 2:**
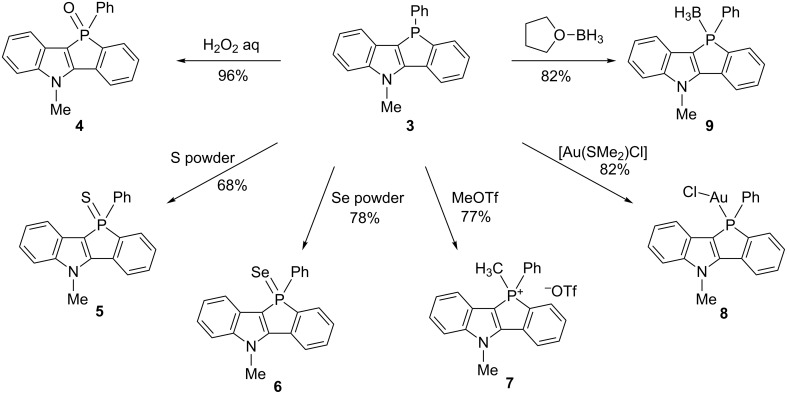
Chemical modifications of the phosphorus atom of **3**.

Then, the chemical modification of the phosphorus atom of **3** was carried out and the results are shown in Scheme 2. The treatment of **3** with hydrogen peroxide, elemental sulfur, and elemental selenium afforded the corresponding phosphine oxide **4**, sulfide **5**, and selenide **6**, respectively. The reaction of **3** with methyl triflate afforded phospholium triflate **7**. Phosphole **3** was treated with chloro(dimethyl sulfide)gold in CH_2_Cl_2_, resulting in P-complexation and thus affording the gold complex **8**. The borane complex **9** was readily prepared from **3** by treating with borane in THF.

**Figure 2:**
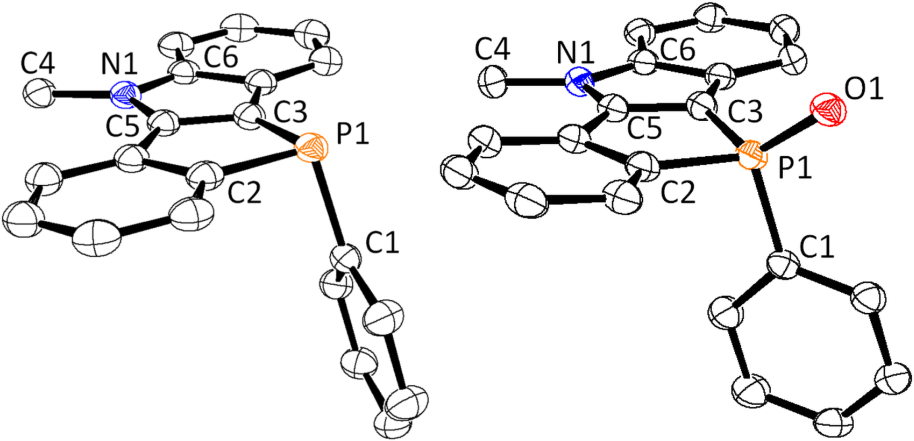
ORTEP drawing of compound **3** (left) and **4** (right) with 50% probability. All hydrogen atoms are omitted for clarity. One of two geometries in the unit cell was drawn for **4**.

The molecular structures of compounds **3**–**9** were confirmed by elemental and spectral analyses (^1^H, ^13^C, and ^31^P NMR, MS, and IR). The ^31^P NMR spectra show the typical low-field shift (**4**: δ = 22.0, **5**: δ = 27.0, **6**: δ = 11.0, **7**: δ = 6.0 ppm) relative to the parent compound **3** (δ = −29.1 ppm). The corresponding ^31^P NMR signals of gold and boron complexes **8** and **9** were observed at δ = 6.0 and 11.5 ppm, respectively. These results show that the electronic nature of the phosphorus atoms is similar to that of the oxidized species **4**–**7** (δ = 6.0–27.0 ppm). In the IR spectrum of **4** in KBr, a strong absorption for P=O stretching vibration at 1188 cm^−1^ was observed. Figure 2 shows the X-ray crystal structures of the benzophospholo[3,2-*b*]indoles **3** and phosphine oxide **4**. Selected bond lengths and angles are listed in Table 1. Figure 2 clearly shows that the tetracyclic skeletons are planar. The mean deviations are 0.022 Å for **3**, and 0.040 Å and 0.059 Å for two independent molecules in the unit cell of compound **4**, comparable to those of benzophosphole-fused tetracyclic heteroacenes (0.016–0.057 Å) [26,29–30,33]. The sums of the bond angles around the nitrogen atom were 359.98° for **3**, and 360.02° and 359.99° for **4**. In contrast, these angles around the phosphorus atom were 291.05° for **3**, and 305.46° and 304.75° for **4**. These facts indicate that the nitrogen atoms are sp^2^-hybridized, and the phosphorus center adopts pyramidal for **3** and tetrahedral geometry for **4**.

**Table 1:** Selected bond length and angles.

	**3**	**4**

Bond length [Å]		

P1–C1	1.8467(12)	1.8108(12)
P1–C2	1.8459(14)	1.8215(13)
P1–C3	1.7958(12)	1.7668(12)
P1–O1		1.4897(9)
N1–C4	1.4533(16)	1.4642(16)
N1–C5	1.3765(14)	1.3679(16)
N1–C6	1.3869(17)	1.388(2)

Bond angles [º]		

C4–N–C5	127.05(11)	127.33(11)
C4–N–C6	125.16(10)	124.71(12)
C5–N–C6	107.77(10)	107.77(11)
C1–P–C2	99.13(5)	106.78(6)
C1–P–C3	102.94(5)	107.01(6)
C2–P–C3	88.97(6)	91.67(6)
O1–P1–C3		121.14(6)
O1–P1–C1		110.64(6)
O1–P1–C2		117.52(6)

**Figure 3:**
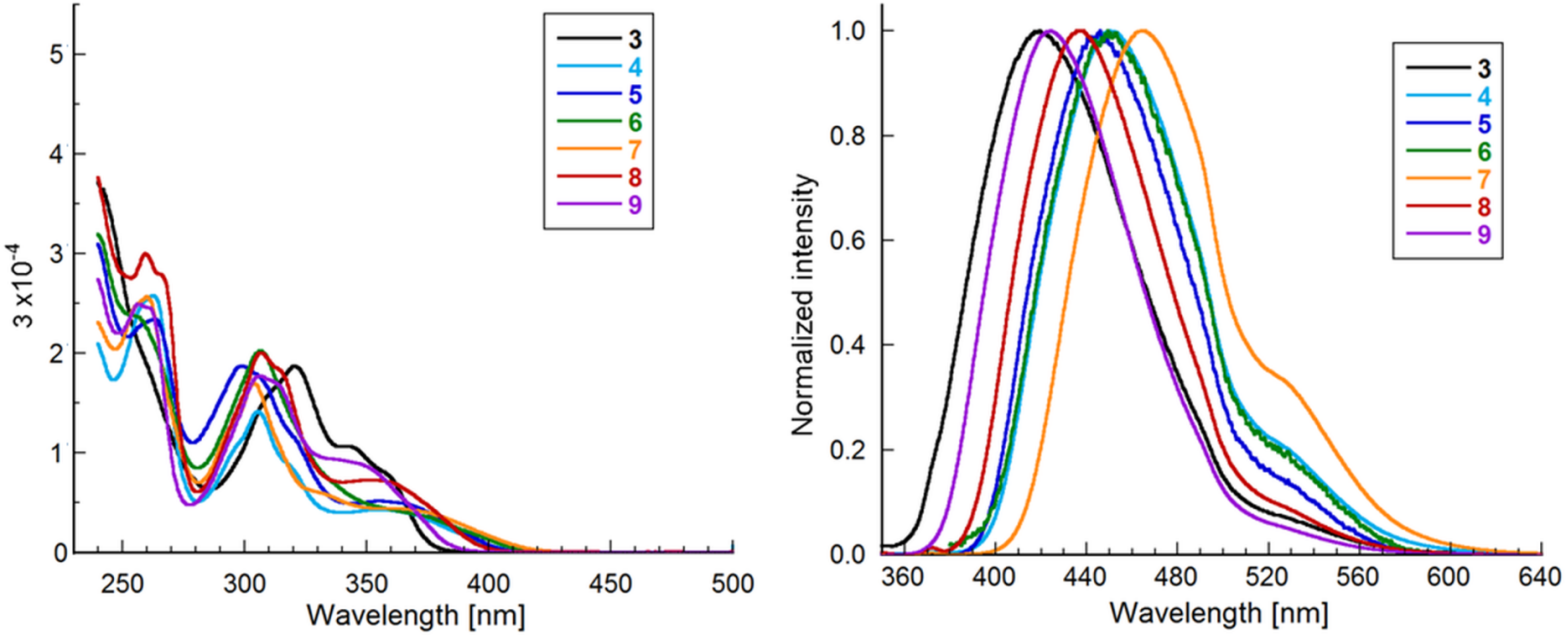
UV–vis absorption (left) and normalized fluorescence emission (right, excitation at 335 nm) spectra in CH_2_Cl_2_.

The photophysical properties of the benzophospholo[3,2-*b*]indoles were evaluated using UV absorption and fluorescence spectroscopy in CH_2_Cl_2_. The spectra are shown in Figure 3, and the photophysical data are shown in Table 2. The functionalized phosphole derivatives **4**–**8** showed absorption maxima (λ_abs_) at 299–307 nm and a broad absorption at ≈355 nm. In contrast, parent phosphole **3** showed narrow absorption peaks at 320, 343, and 357 nm. Additionally, these compounds exhibited very little solvent dependence (see Supporting Information File 1, Figure S2). Phosphine oxide **4** exhibited blue fluorescence with the maximum emission (λ_em_) at 450 nm. The quantum yield (Ф = 75%) was high, comparable to that of phosphole and indole-fused pentacyclic heteroacene (Ф = 70%) [35]. On the other hand, a low fluorescence intensity was observed for phosphine sulfide **5** and selenide **6** (Ф = 1% and 0.3%, respectively). Quenching of fluorescence emission due to a soft sulfur substituent has been reported for several phosphine sulfide (P=S) compounds [10,15,17,20]. The cationic phospholium **7** exhibited green fluorescence (λ_em_ = 465 nm) with the largest red shift of this series of phospholes owing to the cationic nature of the phosphorus atom, providing particularly strong electron-accepting properties. This red shift related to methylation of a phosphorus atom is in line with other earlier studies [10,18,33]. The fluorescence intensity of **7** (Ф = 67%) was as strong as that of phosphine oxide **4**. The gold and boron complexes (**8** and **9**, respectively) showed contrasting fluorescence properties with respect to the intensity. Complex **9** exhibited a high quantum yield (Ф = 75%), while complex **8** exhibited a weak emission (Ф = 11%). In these fluorescence spectra of compounds **4**–**7**, a vibronic band was detected as a shoulder peak around 530 nm. In the case of phosphonium cation **7**, the corresponding vibronic band was seen in the absorption spectrum at 330–340 nm.

**Table 2:** Absorption and fluorescence spectroscopy data.^a^

	λ_max_ [nm]		λ_em_ [nm]^b^	Ф [%]^b^

**3**	321	343	420	5.3%
**4**	306	355^c^	450	75%
**5**	299	355^c^	446	1.0%
**6**	307		450	0.3%
**7**	304		465	67%
**8**	307	355^c^	437	11%
**9**	306	350^c^	425	75%

^a^In CH_2_Cl_2_. ^b^Excitation at 335 nm, and quantum yield using anthracene as standard. ^c^Broad peak.

**Figure 4:**
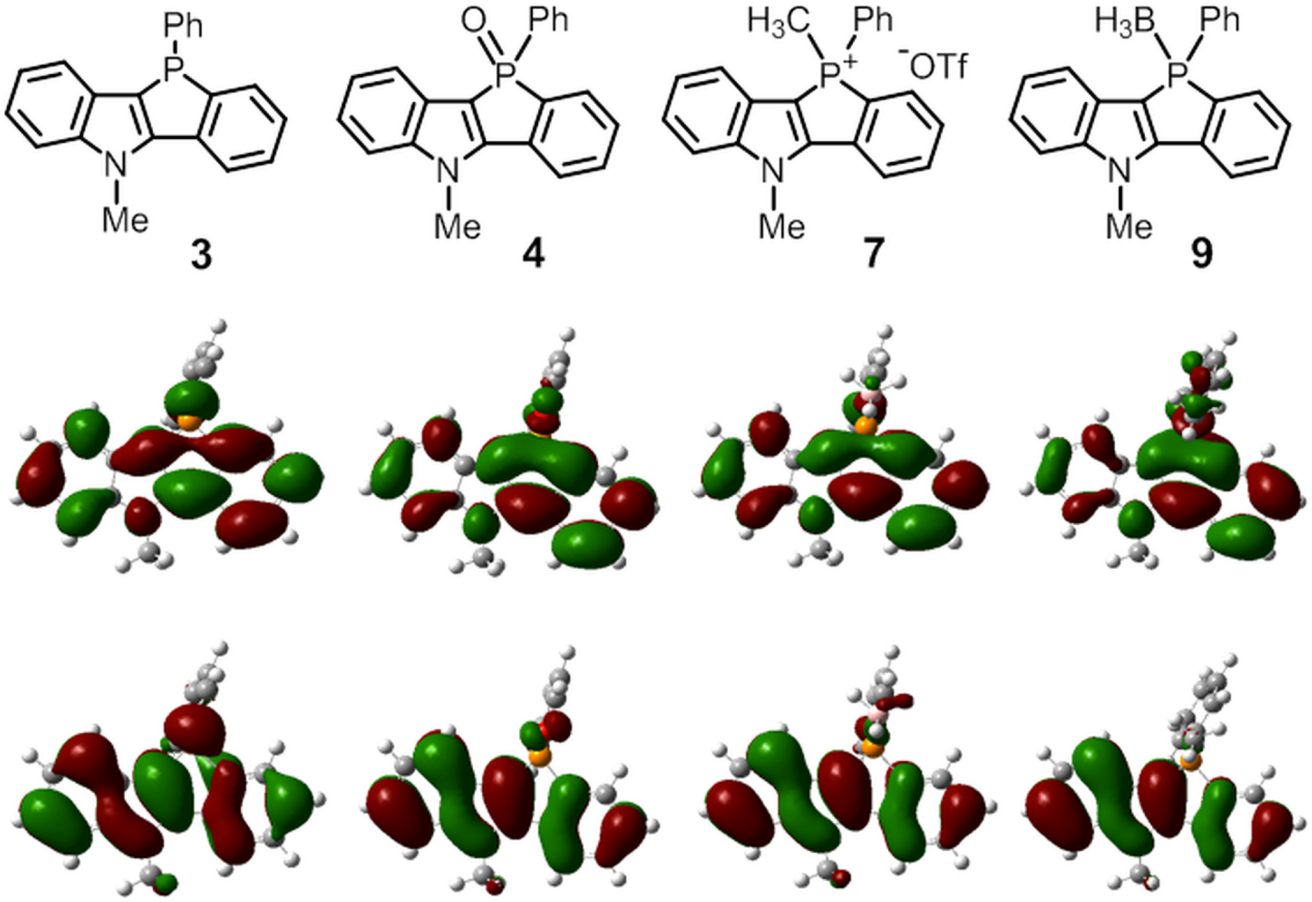
The spatial plots of the HOMO and LUMO of compounds **3**, **4**, **7** and **9**. The calculations were performed at the level of B3LYP/LanL2DZ.

**Figure 5:**
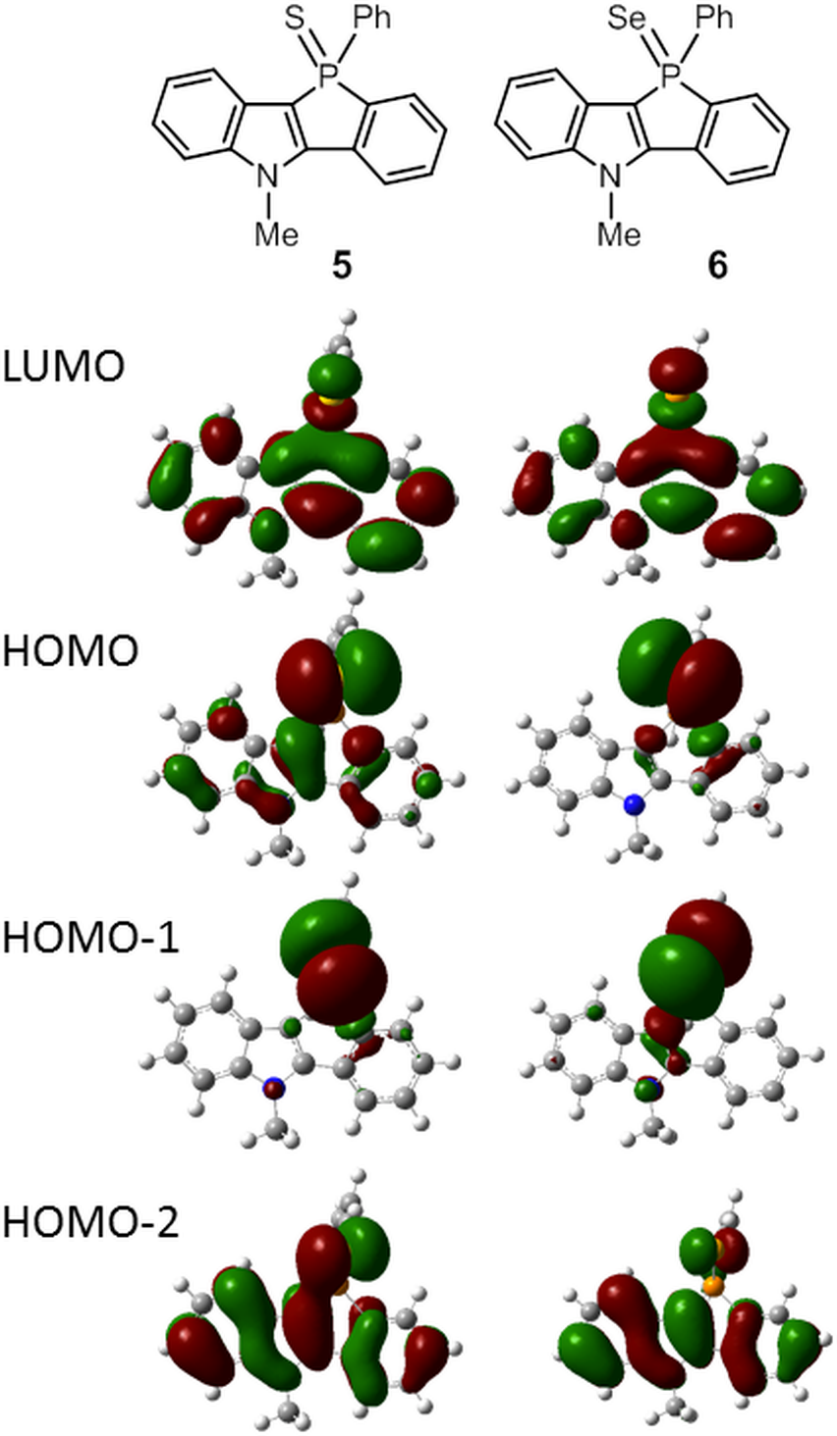
The spatial plots of the selected molecular orbitals of compounds **5** and **6**. The calculations were performed at the level of B3LYP/LanL2DZ.

Density functional theory (DFT) calculations [38] were carried out at the B3LYP/LanL2DZ level of theory. The HOMO and LUMO energies of the selected compounds are given in Table 3. For fluorescent compounds **3**, **4**, **7**, and **9**, the HOMO and LUMO correspond to the π and π* orbitals of the benzophospholoindole skeletons, respectively (Figure 4). Both the HOMO and LUMO energy levels in the functionalized phosphole derivatives **4** and **9** are lower than the parent phosphole **3** owing to the increased electron deficiency of the phosphorus center. Because of the cationic nature of the phosphorus center, the energy levels in cationic phospholium **7** are significantly stabilized. In contrast to the fluorescent phospholes, calculations show that the HOMO and HOMO−1 of nonfluorescent phosphole sulfide **5** and selenide **6** have a large contribution from the lone-pair orbitals on the S and Se atoms, respectively, and the HOMO−2 is delocalized over the conjugated π-system (Figure 5). According to the time-dependent DFT calculations for **5** and **6**, the S_0_ → S_1_ transitions are mainly dominated by the dipole-forbidden HOMO–LUMO (lp–π*) transition; this may be associated with the low fluorescence quantum yields.

**Table 3:** Calculated HOMO and LUMO levels of phospholes.

Compound	HOMO [eV]^a^	LUMO [eV]^a^

**3**	−5.34	−1.25
**4**	−5.75	−1.74
**5**	−5.54	−1.78
**6**	−5.27	−1.79
**7**^b^	−8.82	−5.00
**9**	−5.70	−1.62

^a^DFT calculation at the level of B3LYP/LanL2DZ. ^b^Cation part only.

## Conclusion

A series of novel indole-fused phospholes were synthesized by simple chemical modifications at the trivalent phosphorus center. These organophosphorus compounds generated a whole series of derivatives from only one precursor. The X-ray crystal analysis of benzophospholo[3,2-*b*]indoles showed that the nitrogen atoms are sp^2^ hybridized and the phosphorus atoms adopt pyramidal and tetrahedral geometry. A significant characteristic of the benzophosphole-fused indole derivatives is that the corresponding phosphine oxide, the phospholium salt, and the borane complex showed a high fluorescence emission. Further investigations are underway to develop functional materials including electronic devices and evaluate the physicochemical properties of these compounds by synthetic, theoretical, and spectroscopic studies.

## Supporting Information

File 1 Experimental details, characterization data, and NMR spectra of all new compounds.

## References

[1] Aitken R A (2001). Sci Synth.

[2] Mathey F (2002). Sci Synth.

[3] Hissler M, Dyer P W, Réau R (2003). Coord Chem Rev.

[4] Baumgartner T, Réau R (2006). Chem Rev.

[5] Hobbs M G, Baumgartner T (2007). Eur J Inorg Chem.

[6] Matano Y, Imahori H (2009). Org Biomol Chem.

[7] Matano Y, Nakabuchi T, Imahori H (2010). Pure Appl Chem.

[8] Zagidullin A A, Bezkishko I A, Miluykov V A, Sinyashin O G (2013). Mendeleev Commun.

[9] Duffy M P, Delaunay W, Bouit P-A, Hissler M (2016). Chem Soc Rev.

[10] Chan J C-H, Lam W H, Wong H-L, Wong W-T, Yam V W-W (2013). Angew Chem, Int Ed.

[11] Ren Y, Baumgartner T (2010). Chem – Asian J.

[12] Dienes Y, Durben S, Kárpáti T, Neumann T, Englert U, Nyulászi L, Baumgartner T (2007). Chem – Eur J.

[13] Durben S, Dienes Y, Baumgartner T (2006). Org Lett.

[14] Dienes Y, Eggenstein M, Neumann T, Englert U, Baumgartner T (2006). Dalton Trans.

[15] Baumgartner T, Bergmans W, Kárpáti T, Neumann T, Nieger M, Nyulászi L (2005). Chem – Eur J.

[16] Baumgartner T, Neumann T, Wirges B (2004). Angew Chem, Int Ed.

[17] He X, Woo A Y Y, Borau-Garcia J, Baumgartner T (2013). Chem – Eur J.

[18] Dienes Y, Eggenstein M, Kárpáti T, Sutherland T C, Nyulászi L, Baumgartner T (2008). Chem – Eur J.

[19] Fukazawa A, Yamaguchi S (2009). Chem – Asian J.

[20] Fukazawa A, Ichihashi Y, Kosaka Y, Yamaguchi S (2009). Chem – Asian J.

[21] Wang C, Fukazawa A, Taki M, Sato Y, Higashiyama T, Yamaguchi S (2015). Angew Chem, Int Ed.

[22] Matano Y, Motegi Y, Kawatsu S, Kimura Y (2015). J Org Chem.

[23] Hibner-Kulicka P, Joule J A, Skalik J, Bałczewski P (2017). RSC Adv.

[24] Adler R A, Wang C, Fukazawa A, Yamaguchi S (2017). Inorg Chem.

[25] Fukazawa A, Murai T, Li L, Chen Y, Yamaguchi S (2010). C R Chim.

[26] Fukazawa A, Hara M, Okamoto T, Son E-C, Xu C H, Tamao K, Yamaguchi S (2008). Org Lett.

[27] Fukazawa A, Yamaguchi E, Ito E, Yamada H, Wang J, Irle S, Yamaguchi S (2011). Organometallics.

[28] Fukazawa A, Yamada H, Sasaki Y, Akiyama S, Yamaguchi S (2010). Chem – Asian J.

[29] Fukazawa A, Yamada H, Yamaguchi S (2008). Angew Chem, Int Ed.

[30] Xu Y, Wang Z, Gan Z, Xi Q, Duan Z, Mathey F (2015). Org Lett.

[31] Takahashi M, Nakano K, Nozaki K (2015). J Org Chem.

[32] Weymiens W, Zaal M, Slootweg J C, Ehlers A W, Lammertsma K (2011). Inorg Chem.

[33] Ren Y, Baumgartner T (2011). J Am Chem Soc.

[34] Ren Y, Biegger F, Baumgartner T (2013). J Phys Chem C.

[35] Gong P, Ye K, Sun J, Chen P, Xue P, Yang H, Lu R (2015). RSC Adv.

[36] Matsumura M, Muranaka A, Kurihara R, Kanai M, Yoshida K, Kakusawa N, Hashizume D, Uchiyama M, Yasuike S (2016). Tetrahedron.

[37] Mehta S, Waldo J P, Larock R C (2009). J Org Chem.

[38] (2009). Gaussian 09.

