# Supplementary material for: Synthesis and photophysical properties of novel benzophospholo[3,2-b]indole derivatives
Source: Beilstein J Org Chem. 2017 Oct 30;13:2304–9. doi: 10.3762/bjoc.13.226 (PMC5687007; doi:10.3762/bjoc.13.226)

Supporting Information
for
Synthesis and photophysical properties of novel benzophospholo[3,2-*b*]indole derivatives

Mio Matsumura[1], Mizuki Yamada[1], Atsuya Muranaka*[2], Misae Kanai[2], Naoki Kakusawa[3], Daisuke Hashizume[4], Masanobu Uchiyama[2,5] and Shuji Yasuike*[1]

Address: [1]School of Pharmaceutical Sciences, Aichi Gakuin University, 1-100 Kusumoto-cho, Chikusa-ku, Nagoya 464-8650, Japan, [2]Elements Chemistry Laboratory, RIKEN, and Advanced Elements Chemistry Research Team, RIKEN Center for Sustainable Resource Science (CSRS), Wako 351-0198, Japan, [3]Faculty of Pharmaceutical Sciences, Hokuriku University, Ho-3 Kanagawa-machi, Kanazawa 920-1181, Japan. [4]Materials Characterization Support Unit, RIKEN Center for Emergent Matter Science (CEMS), Wako 351-0198, Japan and [5]Graduate School of Pharmaceutical Sciences, The University of Tokyo, Tokyo 113-0033, Japan

Email: Atsuya Muranaka - atsuya-muranaka@riken.jp;

Shuji Yasuike - s-yasuik@dpc.agu.ac.jp

* Corresponding author

Experimental details, characterization data, and NMR spectra of all new compounds

Contents

1. General information S3

2. Experimental details and characterization data S3

3. X-ray crystal structure determinations of **3** and **4** S9

4. Absorption and fluorescence spectra S10

5. Computational details S10

6. References S15

7. NMR spectra of new compounds S16

1. General information

Melting points measurements were conducted on a Yanagimoto micro melting point hot-stage apparatus (MP-S3) and reported as uncorrected values. ^{1}H NMR (TMS: δ 0.00 as an internal standard), ^{13}C NMR ($CDCl_3$: δ 77.0 as an internal standard), and ^{31}P NMR (85% H_3PO_4: δ 0.0 as an external standard) spectra were recorded on JEOL JNM-AL400 (for ^{1}H and ^{13}C NMR, 400 MHz and 100 MHz, respectively) and JNM-ECA500 (for ^{31}P NMR, 202 MHz) spectrometers in $CDCl_3$. Mass spectra were obtained on a JEOL JMP-DX300 instrument (70 eV, 300 μA). IR spectra were recorded on a FTIR-8400S system from Shimadzu spectrometer and were reported in frequency of absorption (cm^{-1}). Only selected IR absorbancies are reported. UV–vis spectra were recorded at room temperature on a HITACHI U-2800A spectrophotometer and fluorescence spectra on a JASCO FP-8300 luminescence spectrometer.

All chromatographic separations were accomplished with Silica Gel 60N (Kanto Chemical Co., Inc.). Thin-layer chromatography (TLC) was performed using Macherey-Nagel Pre-coated TLC plates Sil G25 UV_{254}.

2. Experimental details and characterization data

2-[(2-Bromophenyl)ethynyl]-*N*,*N*-dimethylaniline (**1**) [1]

To a solution of 2-iodo-*N*,*N*-dimethylaniline (1.48 g, 6 mmol) in triethylamine (25 mL), $PdCl_2(PPh_3)_2$ (71 mg, 0.1 mmol, 2.0 mol %) and CuI (10 mg, 0.05 mmol, 1.0 mol %) were added, and the mixture was stirred at 0 °C for 20 mim. To the reaction mixture, 1-bromo-2-ethynylbenzene (906 mg, 5 mmol) was added and stirred at 0 °C for 10 min. The reaction mixture was stirred at room temperature for 18 h, and then concentrated under reduced pressure. The residue was diluted with CH_2Cl_2 (150 mL) and water (100 mL). The aqueous phase was extracted with CH_2Cl_2 (150 mL × 2). The combined extracts were washed with water (100 mL × 2) and brine (100 mL × 2) and dried over anhydrous magnesium sulfate, filtered, and concentrated under reduced pressure. The residue was purified by column chromatography using hexane/CH_2Cl_2 (5:1) as eluent to give **1** as brown oil (1.15 g, 77% yield). ^{1}H NMR (400 MHz, $CDCl_3$) *δ*: 3.03 (s, 6H, *N*-CH_3), 6.90 (t, *J* = 7.3 Hz, 2H, Ar-H), 7.16 (t, *J* = 7.7 Hz, 1H, Ar-H), 7.25-7.30 (m, 2H, Ar-H), 7.55-7.57 (m, 2H, Ar-H), 7.60 (d, *J* = 7.8 Hz, 1H, Ar-H). ^{13}C NMR (100 MHz, $CDCl_3$) *δ*: 43.7 (CH_3), 93.1 (C), 93.5 (C), 114.6 (C), 116.9 (CH), 120.3 (CH), 125.2 (C), 126.1 (C), 126.9 (CH), 129.0 (CH), 129.7 (CH), 132.4 (CH), 133.2 (CH), 134.8 (CH), 154.7 (C). FTIR (KBr): 2210, 1593,

1495, 1429, 1330 cm^{-1}. LRMS (EI) m/z: 300 ($[M+H]^+$, 100%), 218 (87%), 204 (80%), 144 (80%), 120 (77%). HRMS: m/z $[M]^+$ calcd for $C_{16}H_{14}BrN$: 299.0310. Found: 299.0319.

2-(2-Bromophenyl)-3-iodo-*N*-methylindole (**2**)

To a solution of 2-[(2-bromophenyl)ethynyl]-*N*,*N*-dimethylaniline (**1**) (2.10 g, 7 mmol) in CH_2Cl_2 (25 mL), a solution of I_2 (3.57 g, 14 mmol, 2 equiv) in CH_2Cl_2 (70 mL) was added dropwise, and the mixture was stirred at room temperature for 1 h. The reaction mixture was quenched with saturated aqueous sodium thiosulfate solution (100 mL) at 0 °C. The aqueous phase was extracted with CH_2Cl_2 (150 mL x 2). The combined extracts were washed with water (100 mL) and brine (100 mL), dried over anhydrous magnesium sulfate, filtered, and concentrated under reduced pressure. The residue was purified by column chromatography using hexane/CH_2Cl_2 (2:1) as eluent to give **2** as pale yellow prism (2.82 g, 98% yield), mp 133-133.5 °C (from CH_2Cl_2-hexane). ^{1}H NMR (400 MHz, $CDCl_3$) δ: 3.60 (s, 3H, *N*-CH_3), 7.22-7.39 (m, 5H, Ar-H), 7.46 (t, J = 7.3 Hz, 1H, Ar-H), 7.51 (d, J = 7.8 Hz, 1H, Ar-H), 7.75 (d, J = 8.0 Hz, 1H, Ar-H). ^{13}C NMR (100 MHz, $CDCl_3$) δ: 31.6 (CH_3), 59.5 (C), 109.8 (CH), 120.7 (CH), 121.4 (CH), 123.0 (CH), 125.5 (C), 127.4 (CH), 130.0 (C), 130.9 (CH), 132.9 (CH), 133.3 (CH), 133.5 (C), 137.2 (C), 141.0 (C). LRMS (EI) m/z: 411 (M^+, 100%), 204 (75%), 102 (20%). HRMS: m/z $[M]^+$ calcd for $C_{15}H_{11}BrIN$: 410.9120. Found: 410.9111.

10-Phenyl-[1]benzophospholo[3,2-*b*]-*N*-methylindole (**3**)

To a solution of 2-(2-bromophenyl)-3-iodo-*N*-methylindole (**2**, 825 mg, 2 mmol) in dry diethyl ether (40 mL), *n*-BuLi (1.60 M in hexane, 5.0 mL, 8 mmol, 4 equiv) was added and the mixture was stirred at −78 °C for 15 min. To the reaction mixture, $PhPCl_2$ (0.8 mL, 6 mmol, 3 equiv) was added and the mixture was raised gradually to room temperature. After 17 h, the reaction mixture was diluted with CH_2Cl_2 (150 mL) and water (100 mL). The aqueous phase was extracted with CH_2Cl_2 (150 mL × 2). The combined extracts were washed with brine (100 mL × 2), dried over anhydrous magnesium sulfate, filtered, and concentrated under reduced pressure. The residue was purified by column chromatography using hexane/CH_2Cl_2 (2:1) as eluent to give **3** as colorless prism (415 mg, 66% yield), mp 108–109 °C (from hexane-CH_2Cl_2). ^{1}H NMR (400 MHz, $CDCl_3$) δ: 4.11 (s, 3H, *N*-CH_3), 7.15 (t, J = 7.6 Hz, 1H, Ar-H), 7.22-7.30 (m, 5H, Ar-H), 7.36-7.46 (m, 4H, Ar-H), 7.64 (d, J = 7.8 Hz, 1H,

Ar-H), 7.71 (t, J = 6.6 Hz, 1H, Ar-H), 7.87 (d, J = 7.8 Hz, 1H, Ar-H). ^{13}C NMR (100 MHz, $CDCl_3$) δ: 31.6 (q), 109.8 (CH), 114.1 (C), 120.2 (CH), 120.4 (CH) 120.5 (CH), 122.3 (CH), 126.0 (CH, $J_{C,P}$ = 8.3 Hz), 127.3 (C, $J_{C,P}$ = 15 Hz), 128.1 (CH), 128.6 (CH, $J_{C,P}$ = 7.4 Hz), 128.9 (CH), 131.1 (CH, $J_{C,P}$ = 22 Hz), 132.1 (CH, $J_{C,P}$ = 20 Hz), 135.5 (C), 136.4 (C, $J_{C,P}$ = 19 Hz), 142.4 (C, $J_{C,P}$ = 4.1 Hz), 147.4 (C), 148.7 (C, $J_{C,P}$ = 6.6 Hz). ^{31}P{^{1}H} NMR (202 MHz, $CDCl_3$) δ: -29.0 (s). LRMS (EI) m/z: 313 (M^+, 100%), 298 (20%), 236 (45%). HRMS: m/z $[M]^+$ calcd for $C_{21}H_{16}NP$: 313.1020. Found: 313.1028.

10-Phenyl-10-oxo-[1]benzophospholo[3,2-*b*]-*N*-methylindole (**4**)

To a solution of 10-phenyl-[1]benzophospholo[3,2-*b*]-*N*-methylindole (**3**, 418 mg, 1.3 mmol) in dry dichloromethane (70 mL), hydrogen peroxide (30% solution in water, 1.5 mL, 13 mmol, 10 equiv) was added and stirred at 0 °C under air. After 30 min, the mixture was stirred at room temperature for 30 min. The reaction mixture was diluted with CH_2Cl_2 (50 mL) and water (100 mL). The aqueous phase was extracted with CH_2Cl_2 (100 mL × 2). The combined extracts were washed with water (100 mL × 2) and brine (100 mL × 2), dried over anhydrous magnesium sulfate, filtered, and concentrated under reduced pressure. The residue was purified by recrystallization to give **4** as pale yellow prism (420 mg, 96% yield), mp 112.5-113.5 °C (from CH_2Cl_2-hexane). ^{1}H NMR (400 MHz, $CDCl_3$) δ: 4.06 (s, 3H, *N*-CH_3), 7.14 (t, J = 7.3 Hz, 1H, Ar-H), 7.21-7.28 (m, 2H, Ar-H), 7.33-7.39 (m, 3H, Ar-H), 7.41-7.47 (m, 2H, Ar-H), 7.60 (d, J = 7.8 Hz, 1H, Ar-H), 7.64-7.68 (m, 2H, Ar-H), 7.76 (d, J = 8.0 Hz, 1H, Ar-H), 7.79 (d, J = 8.0 Hz, 1H, Ar-H). ^{13}C NMR (100 MHz, $CDCl_3$) δ: 31.9 (CH_3), 106.9 (C, $J_{C,P}$ = 130 Hz), 110.3 (CH), 120.4 (CH, $J_{C,P}$ = 8.3 Hz), 120.8 (CH), 122.2 (CH), 123.3 (CH), 125.6 (C, $J_{C,P}$ = 9.1 Hz), 128.6 (CH, $J_{C,P}$ = 13 Hz), 128.8 (CH), 130.2 (CH, $J_{C,P}$ = 9.1 Hz), 131.1 (CH, $J_{C,P}$ = 12 Hz), 131.5 (C, $J_{C,P}$ = 110 Hz), 131.9 (CH, $J_{C,P}$ = 3.3 Hz), 132.1 (CH, $J_{C,P}$ = 1.7 Hz), 134.9 (C, $J_{C,P}$ = 15 Hz), 140.4 (C, $J_{C,P}$ = 106 Hz), 142.4 (C, $J_{C,P}$ = 11 Hz), 149.8 (C, $J_{C,P}$ = 34 Hz). ^{31}P{^{1}H} NMR (202 MHz, $CDCl_3$) δ: 22.0 (s). FTIR (KBr): 1477, 1410, 1188, 742, 550 cm^{-1}. LRMS (EI) m/z: 329 (M^+, 100%), 281 (45%), 252 (60%). HRMS: m/z $[M]^+$ calcd for $C_{21}H_{16}NOP$: 329.0970. Found: 329.0963.

10-Phenyl-10-thioxo-[1]benzophospholo[3,2-*b*]-*N*-methylindole (**5**)

To a solution of 10-phenyl-[1]benzophospholo[3,2-*b*]-*N*-methylindole (**3**, 313 mg, 1 mmol) in dry benzene (5 mL), elementary sulfur (160 mg, 5 mmol, 5 equiv) was added and stirred at 60 °C. After 3 h, the reaction mixture was

diluted with benzene (20 mL) and water (20 mL). The aqueous phase was extracted with benzene (15 mL × 1). The combined extracts were washed with brine (30 mL × 2), dried over anhydrous magnesium sulfate, filtered, and concentrated under reduced pressure. The residue was purified by column chromatography using hexane/CH_2Cl_2 (2:1) as eluent to give **5** as pale yellow plate (234 mg, 68% yield), mp 243–244 °C (from CH_2Cl_2-hexane). ^1H NMR (400 MHz, $CDCl_3$) δ: 4.02 (s, 3H, *N*-CH_3), 7.14 (td, J = 1.0, 6.8 Hz, 1H, Ar-H), 7.23 (dd, J = 1.5, 7.3 Hz, 1H, Ar-H), 7.27-7.35 (m, 4H, Ar-H), 7.42 (tt, J = 2.0, 7.8 Hz, 2H, Ar-H), 7.63-7.70 (m, 3H, Ar-H), 7.82 (d, J = 6.8 Hz, 1H, Ar-H), 7.86 (d, J = 6.8 Hz, 1H, Ar-H). ^{13}C NMR (100 MHz, $CDCl_3$) δ: 31.9 (CH_3), 108.0 (C, $J_{C,P}$ = 113 Hz), 110.4 (CH), 120.2 (CH), 120.6 (CH, $J_{C,P}$ = 7.5 Hz), 122.2 (CH), 123.4 (CH), 125.1 (C, $J_{C,P}$ = 9.9 Hz), 128.6 (CH, $J_{C,P}$ = 9.9 Hz), 128.8 (CH, $J_{C,P}$ = 12 Hz), 129.9 (CH, $J_{C,P}$ = 9.9 Hz), 130.8 (CH, $J_{C,P}$ = 12 Hz), 131.66 (CH, $J_{C,P}$ = 1.7 Hz), 131.69 (CH, $J_{C,P}$ = 7.5 Hz), 131.9 (C, $J_{C,P}$ = 86 Hz), 134.3 (C, $J_{C,P}$ = 12 Hz), 142.5 (C, $J_{C,P}$ = 9.9 Hz), 142.2 (C, $J_{C,P}$ = 88 Hz), 148.2 (C, $J_{C,P}$ = 30 Hz). $^{31}P\{^1H\}$ NMR (202 MHz, $CDCl_3$) δ: 27.0 (s). FTIR (KBr): 1478, 1410, 1097, 744, 717, 646 cm^{-1}. LRMS (EI) m/z: 345 (M^+, 95%), 313 (25%), 268 (40%), 236 (100%). HRMS: m/z $[M]^+$ calcd for $C_{21}H_{16}NPS$: 345.0741. Found: 345.0732.

10-Phenyl-10-selenoxo-[1]benzophospholo[3,2-*b*]-*N*-methylindole (**6**)

To a solution of 10-phenyl-[1]benzophospholo[3,2-*b*]-*N*-methylindole (**3**, 313 mg, 1 mmol) in dry benzene (5 mL), elementary selenium (396 mg, 5 mmol, 5 equiv) was added and stirred at 60 °C. After 3 h, the reaction mixture was diluted with benzene (20 mL), filtered, and concentrated under reduced pressure. The residue was purified by recrystallization to give **6** as pale yellow prism (305 mg, 78% yield), mp 238.5–241 °C (from CH_2Cl_2-hexane). ^1H NMR (400 MHz, $CDCl_3$) δ: 4.07 (s, 3H, *N*-CH_3), 7.18 (t, J = 7.3 Hz, 1H, Ar-H), 7.24-7.46 (m, 7H, Ar-H), 7.67-7.74 (m, 3H, Ar-H), 7.85 (dd, J = 1.5, 8.0 Hz, 1H, Ar-H), 7.89 (dd, J = 1.5, 8.5 Hz, 1H, Ar-H). ^{13}C NMR (100 MHz, $CDCl_3$) δ: 31.8 (CH3), 106.9 (C, $J_{C,P}$ = 104 Hz), 110.5 (CH), 120.1 (CH), 120.6 (CH, $J_{C,P}$ = 7.4 Hz), 122.2 (CH), 123.5 (CH), 125.2 (C, $J_{C,P}$ = 9.1 Hz), 128.6 (CH, $J_{C,P}$ = 13 Hz), 128.9 (CH, $J_{C,P}$ = 12 Hz), 130.3 (CH, $J_{C,P}$ = 11 Hz), 130.9 (C, $J_{C,P}$ = 76 Hz), 131.3 (CH, $J_{C,P}$ = 12 Hz), 131.6 (CH, $J_{C,P}$ = 2.5 Hz), 131.7 (CH, $J_{C,P}$ = 3.3 Hz), 143.1 (C, $J_{C,P}$ = 12 Hz), 142.5 (C, $J_{C,P}$ = 11 Hz), 143.2 (C, $J_{C,P}$ = 80 Hz), 148.0 (C, $J_{C,P}$ = 28 Hz). $^{31}P\{^1H\}$ NMR (202 MHz, $CDCl_3$) δ: 11.0 (s). FTIR (KBr): 1475, 1094, 746, 576 cm^{-1}. LRMS (EI) m/z: 393 (M^+, 30%), 313 (100%), 236 (95%). HRMS: m/z $[M]^+$ calcd

for $C_{21}H_{16}NPSe$: 393.0186. Found: 393.0169.

10-Methyl-10-phenyl-[1]benzophospholo[3,2-*b*]-*N*-methylindol-10-ium trifluoromethansulfonate (**7**)

To a solution of 10-phenyl-[1]benzophospholo[3,2-*b*]-*N*-methylindole (**3**, 313 mg, 1 mmol) in dry CH_2Cl_2 (15 mL), methyl trifluoromethanesulfonate (132 μL, 1.2 mmol, 1.2 equiv) was added and stirred at room temperature. After 4 h, the reaction mixture was concentrated under reduced pressure. The residue was washed with hexane and purified by recrystallization from benzene to give **7** as yellow prisms (294 mg, 77% yield), mp 96-99 °C (from CH_2Cl_2-benzene). ^{1}H NMR (400 MHz, $CDCl_3$) δ: 2.78 (d, $J_{H,P}$ = 14.6 Hz, 3H, *P*-CH_3), 4.26 (s, 3H, *N*-CH_3), 7.37 (t, *J* = 7.6 Hz, 1H, Ar-H), 7.47 (t, *J* = 7.3 Hz, 1H, Ar-H), 7.56-7.65 (m, 5H, Ar-H), 7.73 (t, *J* = 7.3 Hz, 1H, Ar-H), 7.75 (t, *J* = 7.3 Hz, 1H, Ar-H), 8.00 (dd, *J* = 3.9, 7.8 Hz, 1H, Ar-H), 8.03 (d, *J* = 7.3 Hz, 1H, Ar-H), 8.07 (d, *J* = 7.3 Hz, 1H, Ar-H), 8.44 (dd, *J* = 7.4, 11.7 Hz, 1H, Ar-H). ^{13}C NMR (100 MHz, $CDCl_3$) δ: 8.6 (CH_3, $J_{C,P}$ = 56.3 Hz), 32.6 (CH_3), 91.5 (C, $J_{C,P}$ = 119 Hz), 111.9 (CH), 118.3 (C, $J_{C,P}$ = 89 Hz), 119.5 (CH), 122.2 (CH, $J_{C,P}$ = 8.3 Hz), 124.2 (CH), 124.9 (C, $J_{C,P}$ = 9.9 Hz), 125.1 (CH), 130.0 (C, $J_{C,P}$ = 93 Hz), 130.5 (CH, $J_{C,P}$ = 9.9 Hz), 130.7 (CH, $J_{C,P}$ = 12 Hz), 132.3 (CH, $J_{C,P}$ = 12 Hz), 133.4 (CH, $J_{C,P}$ = 9.9 Hz), 135.1 (C, $J_{C,P}$ = 12 Hz), 135.19 (CH, $J_{C,P}$ = 4.1 Hz), 135.22 (CH, $J_{C,P}$ = 4.9 Hz), 143.2 (C, $J_{C,P}$ = 12 Hz), 152.3 (C, $J_{C,P}$ = 31 Hz). $^{31}P\{^{1}H\}$ NMR (202 MHz, $CDCl_3$) δ: 6.0 (s). LRMS (EI) m/z: 345 (20%), 329 ($[M\text{-}OTf+H]^+$, 100%), 313 (40%). HRMS: m/z $[M\text{-}OTf]^+$ calcd for $C_{22}H_{19}NP$: 328.1250. Found: 328.1261.

Gold complex **8**

To a solution of chloro(dimethyl sulfide)gold(I) (147 mg, 0.5 mmol) in dry CH_2Cl_2 (10 mL), methyl 10-phenyl-[1]benzophospholo[3,2-*b*]-*N*-methylindole (**3**, 157 mg, 0.5 mmol, 1 equiv) was added and stirred at room temperature. After 2 h, the reaction mixture was concentrated under reduced pressure. The residue was washed with hexane and purified by recrystallization to give **8** as colorless plate (223 mg, 82% yield), mp 251–256 °C (from CH_2Cl_2-hexane). ^{1}H NMR (400 MHz, $CDCl_3$) δ: 4.16 (s, 3H, *N*-CH_3), 7.19 (t, *J* = 7.8 Hz, 1H, Ar-H), 7.30-7.37 (m, 4H, Ar-H), 7.42-7.46 (m, 2H, Ar-H), 7.51-7.66 (m, 5H, Ar-H), 7.87 (dd, *J* = 2.4, 7.8 Hz, 1H, Ar-H). ^{13}C NMR (100 MHz, $CDCl_3$) δ: 32.0 (CH_3), 104.4 (C, $J_{C,P}$ = 85 Hz), 110.6 (CH), 120.2 (CH), 121.1 (CH, $J_{C,P}$ = 6.6 Hz), 122.3 (CH), 123.8 (CH), 125.6 (C, $J_{C,P}$ = 12 Hz), 128.1 (CH, $J_{C,P}$ = 12 Hz), 128.8 (C), 129.3 (CH, $J_{C,P}$ =

13 Hz), 131.7 (CH, $J_{C,P}$ = 1.7 Hz), 132.2 (CH, $J_{C,P}$ = 14 Hz), 132.3 (CH, $J_{C,P}$ = 3.3 Hz), 133.3 (CH, $J_{C,P}$ = 16 Hz), 135.2 (C, $J_{C,P}$ = 8.2 Hz), 139.8 (C, $J_{C,P}$ = 65 Hz), 142.5 (C, $J_{C,P}$ = 9.1 Hz), 149.1 (C, $J_{C,P}$ = 22 Hz). $^{31}P\{^{1}H\}$ NMR (202 MHz, $CDCl_3$) δ: 6.0 (s). LRMS (EI) m/z: 510 ($[M-Cl]^+$, 40%), 119 (60%), 85 (100%). HRMS: m/z $[M-Cl]^+$ calcd for $C_{21}H_{16}AuNP$: 510.0686. Found: 510.0663.

Borane complex **9**

To a solution of 10-phenyl-[1]benzophospholo[3,2-*b*]-*N*-methylindole (**3**, 313 mg, 1 mmol) in dry THF (15 mL), borane·THF complex (1.0 M in THF solution, 1.5 mL, 1.5 mmol, 1.5 equiv) was added at 0 °C and stirred. After 15 min, the reaction mixture was stirred at room temperature for 1 h. Then, the reaction mixture was concentrated under reduced pressure. The residue was washed with hexane and purified by recrystallization to give **9** as white powder (270 mg, 82% yield), mp 201–202.5 °C (from CH_2Cl_2-hexane). ^{1}H NMR (400 MHz, $CDCl_3$) δ: 4.07 (s, 3H, *N*-CH_3), 7.17 (td, *J* = 1.0, 7.3 Hz, 1H, Ar-H), 7.24-7.34 (m, 4H, Ar-H), 7.37-7.43 (m, 2H, Ar-H), 7.47 (tt, *J* = 1.2, 7.3 Hz, 1H, Ar-H), 7.59 (dd, *J* = 1.0, 7.8 Hz, 1H, Ar-H), 7.66-7.72 (m, 3H, Ar-H), 7.80 (dd, *J* = 2.0, 7.8 Hz, 1H, Ar-H). ^{13}C NMR (100 MHz, $CDCl_3$) δ: 31.8 (CH_3), 105.1 (C, $J_{C,P}$ = 78 Hz), 110.3 (CH), 120.4 (CH), 120.7 (CH, $J_{C,P}$ = 5.8 Hz), 121.8 (CH), 123.3 (CH), 126.1 (C, $J_{C,P}$ = 11 Hz), 127.9 (CH, $J_{C,P}$ = 9.9 Hz), 128.4 (C, $J_{C,P}$ = 52 Hz), 128.8 (CH, $J_{C,P}$ = 11 Hz), 131.1 (CH, $J_{C,P}$ = 12 Hz), 131.2 (CH, $J_{C,P}$ = 1.7 Hz), 131.4 (CH, $J_{C,P}$ = 2.5 Hz), 131.9 (CH, $J_{C,P}$ = 11 Hz), 135.8 (C, $J_{C,P}$ = 6.6 Hz), 140.7 (C, $J_{C,P}$ = 59 Hz), 142.6 (C, $J_{C,P}$ = 8.3 Hz), 148.7 (C, $J_{C,P}$ = 17 Hz). $^{31}P\{^{1}H\}$ NMR (202 MHz, $CDCl_3$) δ: 11.5 (s, $J_{P,B}$ = 46.5 Hz). LRMS (EI) m/z: 329 (20%), 313 ($[M-BH_3]^+$, 100%), 236 (50%). Anal. Calc. for $C_{21}H_{19}BNP$•0.5 H_2O: C, 75.03; H, 6.00; N, 4.17. Found: C, 75.04; H, 5.82; N, 4.24.

3. X-ray crystal structure determinations of 3 and 4

The X-ray diffraction measurements of compounds **3** and **4** were carried out using a Rigaku AFC-8 with Saturn70 CCD using Mo Kα radiation (λ = 0.71073 Å). The structure was solved by direct methods using SIR2004, followed by successive refinements by the full-matrix least-squares method on F^2 using SHELXL-2013 [2]. All the non-hydrogen atoms were refined anisotropically, whereas the hydrogen atoms were refined as riding models. Crystallographic information files (CIFs) for **3** and **4** can be obtained free of charge from the Cambridge Crystallographic Data Centre (http://www.ccdc.cam.ac.uk), CCDC #1555048 and #1555048 for **3** and **4**, respectively.

Crystal data of **3** (Figure S1a).

$C_{21}H_{16}NP$, M = 313.32, Triclinic, a = 7.7958(2), b = 10.6445(4), c = 11.0573(5) Å, α = 64.4769(19), β = 73.193(2), γ = 85.8959(17)°, V = 791.15(5) Å^3, Space group P-1, Z = 2, D_{calc} = 1.315 Mg/m^3. Crystal size 0.14 x 0.12 x 0.10 mm^3, $2\theta_{max}$ = 30.265°, 16742 reflections measured, 4683 unique (R_{int} = 0.0349), μ (Mo Kα) = 0.172 mm^{-1}. The final R_1 and wR_2 were 0.0430 and 0.0510 ($I > 2\sigma(I)$), for 209 parameters.

Crystal data of **4** (Figure S1b).

$C_{21}H_{16}NOP$, M = 329.32, Triclinic, a = 9.1278(3), b = 11.4273(3), c = 15.6986(5) Å, α = 88.5466(11), β = 78.5887(14), γ = 88.771(2)°, V = 1604.35(9) Å^3, Space group P-1, Z = 4, D_{calc} = 1.363 Mg/m^3. Crystal size 0.17 x 0.11 x 0.11 mm^3, $2\theta_{max}$ = 30.260°, 68240 reflections measured, 9520 unique (R_{int} = 0.0575), μ (Mo Kα) = 0.172 mm^{-1}. The final R_1 and wR_2 were 0.0397 and 0. 0487 ($I > 2\sigma(I)$), for 579 parameters.

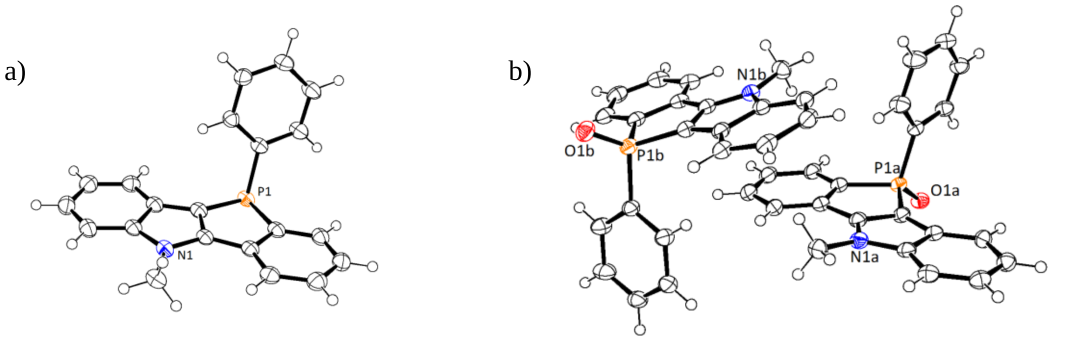

Figure S1: Molecular structures of (a) **3** and (b) **4** with 50% probability thermal ellipsoids.

4. Absorption and fluorescence spectra

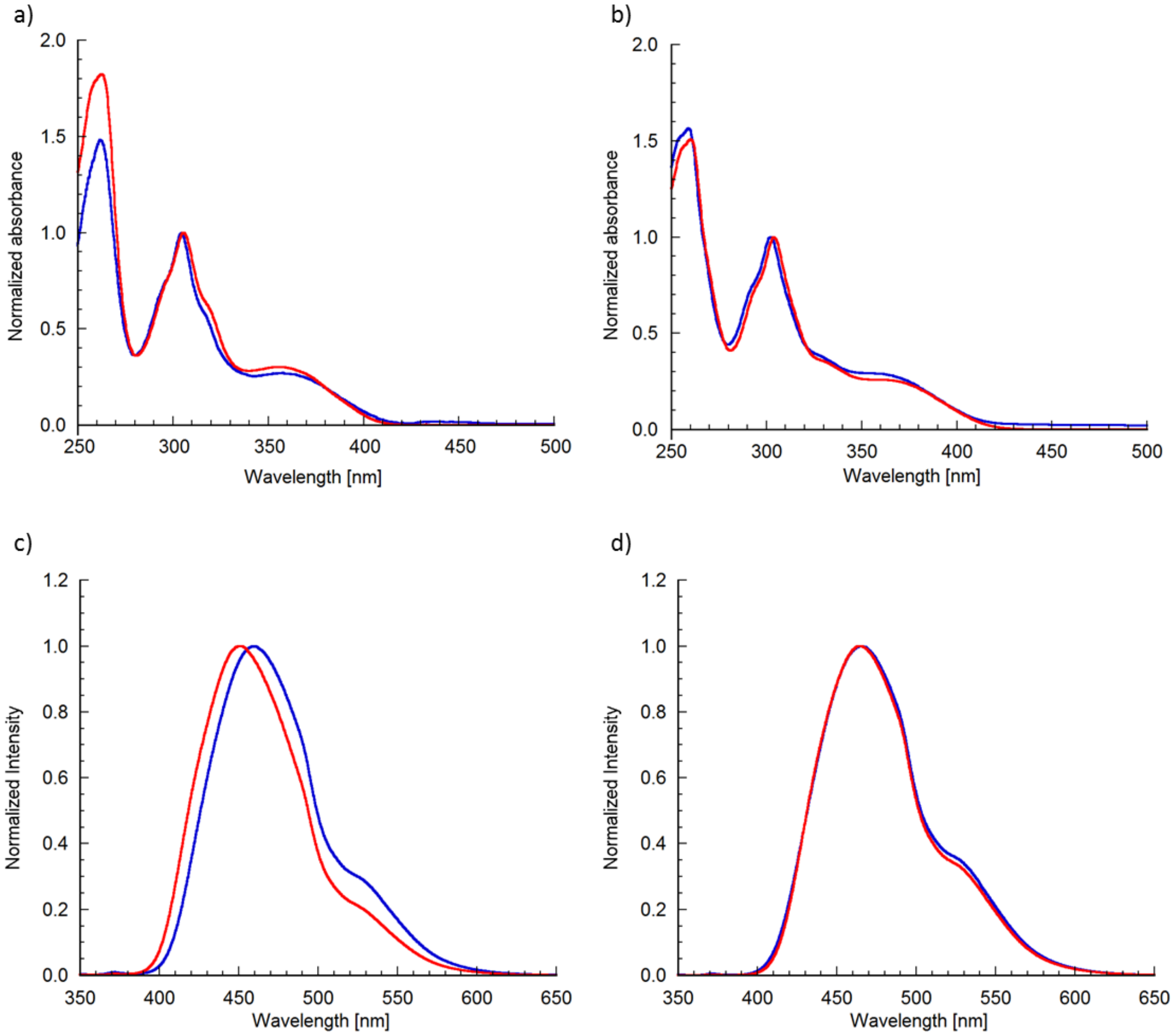

Figure S2: Normalized absorption spectra of (a) **4** and (b) **7**, and fluorescence spectra of (c) **4** and (d) **7**. Red and blue lines show the spectra in CH_2Cl_2 and MeOH, respectively.

5. Computational details

All calculations were performed at the DFT level, by means of the B3LYP functional as implemented in Gaussian 09 [3]. The LanL2DZ basis sets were used. X-ray structures of **3** and **4** were used as initial structures for their geometry optimization calculations. Cationic phospholium **7** was calculated on the cation part only. Calculated excitation energies and oscillator strengths of the optimized structures were obtained using the TDDFT method at the B3LYP/LanL2DZ level.

Table S1: Excitation wavelengths and oscillator strengths.

	Excited state 1		Excited state 2		Excited state 3	
	λ [nm]	*f*	λ [nm]	*f*	λ [nm]	*f*
**3**	335.92	0.1898	301.78	0.1440	299.82	0.0901
**4**	353.60	0.1191	312.59	0.0106	308.01	0.0853
**5**	409.58	0.0014	398.00	0.0134	346.82	0.1243
**6**	452.03	0.0003	439.84	0.0128	352.39	0.0744
**7**	376.67	0.0789	331.95	0.1366	317.32	0.0274
**9**	338.41	0.1792	302.87	0.1064	297.90	0.0346

Cartesian coordinates and energy of **3**

E(B3LYP) = -870.98066704 A.U.

C	0.121361	2.059093	-0.027637
C	1.268773	1.51767	-0.690452
C	2.442642	2.269093	-0.800172
C	2.499279	3.56739	-0.245904
C	1.370694	4.107678	0.400554
C	0.181406	3.362424	0.510337
H	3.313208	1.859592	-1.307748
H	3.413455	4.150846	-0.32281
H	1.416014	5.110286	0.819479
H	-0.675292	3.800235	1.013329
C	-3.572101	-2.704342	-0.633257
C	-4.512456	-1.882562	0.045273
C	-2.279635	-2.241265	-0.912307
H	-3.865194	-3.706079	-0.937974
C	-4.179838	-0.582198	0.45584
H	-5.508138	-2.26894	0.250343
C	-1.915234	-0.934484	-0.510859
H	-1.564995	-2.875439	-1.430892
C	-2.882175	-0.115345	0.173952
H	-4.912452	0.030524	0.974559
C	-0.718237	-0.14827	-0.61193
C	-0.975493	1.091241	-0.020615
N	-2.2877	1.128092	0.461075
C	-2.943429	2.256231	1.121769
H	-2.45396	2.499333	2.073378
H	-2.93657	3.145397	0.47961
H	-3.984159	1.99822	1.330645
C	1.980355	-1.299144	-0.236056
C	3.156065	-1.886158	-0.751819
C	1.621376	-1.539517	1.107181
C	3.973092	-2.68929	0.068887
H	3.429739	-1.724284	-1.792746
C	2.432271	-2.347917	1.924994
H	0.710884	-1.100624	1.507328
C	3.611946	-2.921743	1.409189
H	4.877914	-3.134754	-0.338449
H	2.145683	-2.529265	2.958539
H	4.237585	-3.546374	2.042922
P	0.952414	-0.215789	-1.413923

Cartesian coordinates and energy of **4**

E(B3LYP) = -946.17592991 A.U.

C	0.145848	2.12763	-0.067668	C	0.751283	-0.17854	0.491461
C	-1.084261	1.695104	0.511897	C	1.14242	1.042922	-0.059081
C	-2.168608	2.559872	0.639036	N	2.461189	0.96024	-0.496866
C	-2.050599	3.889378	0.169168	C	3.244935	2.027567	-1.122078
C	-0.846805	4.323183	-0.413264	H	2.771018	2.373044	-2.04855
C	0.255892	3.450834	-0.533972	H	3.36633	2.878309	-0.440631
H	-3.093459	2.220618	1.098787	H	4.237636	1.6467	-1.370875
H	-2.888159	4.575652	0.261924	C	-2.062023	-1.130128	-0.023279
H	-0.759114	5.346037	-0.771772	C	-3.091919	-1.872914	0.585617
H	1.174781	3.816331	-0.980613	C	-1.871157	-1.184331	-1.41767
C	3.335749	-2.9976	0.555576	C	-3.940298	-2.668368	-0.207957
C	4.375896	-2.25604	-0.068314	H	-3.205554	-1.823112	1.666002
C	2.083344	-2.420898	0.799633	C	-2.721902	-1.979227	-2.207396
H	3.521785	-4.027744	0.848634	H	-1.065836	-0.621435	-1.884982
C	4.186029	-0.922093	-0.459413	C	-3.757484	-2.720446	-1.60312
H	5.337962	-2.731545	-0.243395	H	-4.735309	-3.244788	0.258919
C	1.863192	-1.078345	0.413374	H	-2.577025	-2.023977	-3.28414
H	1.294524	-2.989461	1.2844	H	-4.412813	-3.335945	-2.215354
C	2.927351	-0.341693	-0.214867	P	-0.980062	-0.097937	1.099838
H	4.99462	-0.370942	-0.931916	O	-1.348759	-0.391947	2.640994

Cartesian coordinates and energy of **5**

E(B3LYP) = -881.08046507 A.U.

C	0.187543	2.140714	-0.16703	H	1.218811	3.855023	-1.026934
C	-1.054287	1.685812	0.371261	C	3.410662	-2.955322	0.491607
C	-2.155579	2.53222	0.473024	C	4.465378	-2.192039	-0.079474
C	-2.042562	3.866977	0.019153	C	2.142191	-2.397699	0.693144
C	-0.826976	4.326031	-0.519398	H	3.598086	-3.987078	0.777938
C	0.29143	3.472192	-0.613314	C	4.275351	-0.854308	-0.457457
H	-3.086541	2.175024	0.90571	H	5.439369	-2.653569	-0.223223
H	-2.893754	4.538671	0.091996	C	1.921983	-1.052319	0.317324
H	-0.743798	5.354004	-0.863861	H	1.341814	-2.981129	1.139304

C	3.001315	-0.291627	-0.254114
H	5.09535	-0.286669	-0.888926
C	0.798747	-0.165666	0.364083
C	1.19577	1.071263	-0.146773
N	2.533242	1.011053	-0.529364
C	3.331581	2.101522	-1.092907
H	2.914798	2.443946	-2.047828
H	3.384865	2.948693	-0.398667
H	4.348415	1.746881	-1.273646
C	-1.979274	-1.120731	-0.285538
C	-3.089694	-1.837013	0.195787
C	-1.649952	-1.151214	-1.656124
C	-3.881459	-2.58131	-0.700144
H	-3.314888	-1.814944	1.259579
C	-2.443832	-1.895583	-2.547276
H	-0.781322	-0.610929	-2.025243
C	-3.561477	-2.610069	-2.070726
H	-4.738869	-3.13673	-0.32805
H	-2.190365	-1.921781	-3.604338
H	-4.172594	-3.18689	-2.761164
P	-0.946853	-0.118317	0.922271
S	-1.435652	-0.521008	2.945512

Cartesian coordinates and energy of **6**

E(B3LYP) = -880.20445157 A.U.

C	0.455046	2.197229	-0.255541
C	-0.85575	1.765465	0.111353
C	-1.927281	2.654103	0.156099
C	-1.712579	4.009611	-0.18504
C	-0.42774	4.447036	-0.554736
C	0.659675	3.550221	-0.589189
H	-2.913236	2.313194	0.461217
H	-2.539694	4.713858	-0.154873
H	-0.266777	5.491098	-0.812238
H	1.641615	3.916876	-0.870225
C	3.383069	-3.075415	0.383287
C	4.524105	-2.325509	-0.012455
C	2.122426	-2.474306	0.483702
H	3.497148	-4.131485	0.614249
C	4.429648	-0.958309	-0.313211
H	5.489623	-2.820993	-0.080257
C	1.998258	-1.098012	0.183156
H	1.25514	-3.048706	0.796592
C	3.163492	-0.352026	-0.211757
H	5.314487	-0.401863	-0.610102
C	0.912098	-0.165068	0.17042
C	1.411776	1.083529	-0.20468
N	2.780122	0.986021	-0.444019
C	3.677069	2.072326	-0.843485
H	3.369276	2.505016	-1.803008
H	3.701242	2.862295	-0.083219
H	4.689607	1.679776	-0.957964
C	-1.800622	-0.938642	-0.859186
C	-3.004168	-1.610804	-0.581207
C	-1.29007	-0.90553	-2.173199
C	-3.707269	-2.244352	-1.624283
H	-3.371575	-1.644295	0.442032
C	-1.995352	-1.539996	-3.21174
H	-0.350501	-0.401013	-2.385279
C	-3.205887	-2.20848	-2.939164
H	-4.636792	-2.765319	-1.40826
H	-1.601298	-1.516702	-4.224882
H	-3.748459	-2.700337	-3.743274
P	-0.88205	-0.076306	0.539352
Se	-1.665282	-0.620805	2.595956

Cartesian coordinates and energy of **7**

E(B3LYP) = -910.67359191 A.U.

C	0.345871	2.148003	-0.127557	N	2.587894	0.823166	-0.4335
C	-0.952214	1.802754	0.363304	C	3.4944	1.845921	-0.976795
C	-1.997594	2.72324	0.404693	H	3.142655	2.20266	-1.951192
C	-1.758179	4.042797	-0.045911	H	3.585387	2.690583	-0.28546
C	-0.486444	4.40144	-0.525335	H	4.485841	1.411374	-1.111253
C	0.567298	3.463528	-0.571172	C	-2.192045	-1.042756	-0.019517
H	-2.985136	2.446661	0.765252	C	-3.438303	-1.360111	0.562573
H	-2.55881	4.77575	-0.023137	C	-1.864233	-1.498803	-1.314596
H	-0.309902	5.416349	-0.870192	C	-4.361268	-2.136193	-0.161389
H	1.534539	3.771983	-0.951527	H	-3.700151	-1.022141	1.562018
C	3.106089	-3.229258	0.50274	C	-2.794124	-2.273477	-2.029171
C	4.234417	-2.540512	-0.020743	H	-0.899546	-1.26357	-1.756955
C	1.88381	-2.575281	0.693248	C	-4.041283	-2.590574	-1.45531
H	3.200311	-4.281269	0.756629	H	-5.320909	-2.385013	0.28224
C	4.165208	-1.184158	-0.36607	H	-2.54619	-2.630012	-3.024657
H	5.16853	-3.078168	-0.156389	H	-4.756612	-3.190698	-2.010741
C	1.787891	-1.204939	0.354944	P	-0.972608	0.008994	0.893996
H	1.028425	-3.115772	1.090156	C	-1.264367	-0.132209	2.729746
C	2.937346	-0.523708	-0.174811	H	-2.244184	0.276883	2.992477
H	5.038582	-0.678474	-0.76626	H	-1.209201	-1.182028	3.031996
C	0.74865	-0.216829	0.397281	H	-0.486319	0.438602	3.242945
C	1.2635	0.997661	-0.085385				

Cartesian coordinates and energy of **9**

E(B3LYP) = -897.62114705 A.U.

C	0.082553	2.112796	-0.080195	H	-0.94514	5.287219	-0.822597
C	-1.129913	1.639784	0.510732	H	1.045999	3.829329	-1.012039
C	-2.247251	2.466848	0.624322	C	3.419731	-2.911766	0.534279
C	-2.180528	3.791492	0.134719	C	4.439953	-2.138014	-0.083386
C	-0.992776	4.266283	-0.451406	C	2.149946	-2.372467	0.773777
C	0.141048	3.436218	-0.560116	H	3.634541	-3.936704	0.825876
H	-3.159897	2.102497	1.088599	C	4.212605	-0.808847	-0.470814
H	-3.045846	4.443827	0.216902	H	5.416099	-2.58463	-0.256741

C	1.891503	-1.035677	0.390376	C	-3.915108	-2.688091	-0.332645
H	1.376263	-2.966341	1.252791	H	-3.280522	-1.880182	1.579103
C	2.937076	-0.264619	-0.229219	C	-2.588895	-2.005282	-2.261388
H	5.006185	-0.23314	-0.939493	H	-0.93316	-0.674699	-1.846901
C	0.752607	-0.16951	0.465306	C	-3.664893	-2.735316	-1.716625
C	1.111486	1.066109	-0.07738	H	-4.73939	-3.255388	0.092612
N	2.437786	1.025045	-0.504328	H	-2.388904	-2.044589	-3.329486
C	3.197151	2.123316	-1.10432	H	-4.297105	-3.337805	-2.364836
H	2.756145	2.435191	-2.059089	P	-0.964784	-0.138286	1.115485
H	3.239394	2.986148	-0.428893	H	-2.497867	-0.148645	3.201496
H	4.220358	1.792337	-1.294264	H	-1.126162	-1.640603	3.228932
C	-2.024324	-1.175308	-0.038423	H	-0.567013	0.262057	3.65461
C	-3.095796	-1.910122	0.508902	B	-1.333794	-0.459742	3.068251
C	-1.769134	-1.227523	-1.424798				

----

6. References

1. Yan, Y.-Q.; Li, Y.-B.; Wang, J.-W.; Zhao, C.-H. *Chem. -Asian J.*, **2013**, *8*, 3164-3176.

2. Sheldrick, G. M. *Acta Crystallogr. Sect. C*, **2015**, *71*, 3-8.

3. Gaussian 09, Revision E.01, Frisch, M. J.; Trucks, G. W.; Schlegel, H. B.; Scuseria, G. E.; Robb, M. A.; Cheeseman, J. R.; Scalmani, G.; Barone, V.; Mennucci, B.; Petersson, G. A.; Nakatsuji, H.; Caricato, M.; Li, X.; Hratchian, H. P.; Izmaylov, A. F.; Bloino, J.; Zheng, G.; Sonnenberg, J. L.; Hada, M.; Ehara, M.; Toyota, K.; Fukuda, R.; Hasegawa, J.; Ishida, M.; Nakajima, T.; Honda, Y.; Kitao, O.; Nakai, H.; Vreven, T.; Montgomery, J. A., Jr.; Peralta, J. E.; Ogliaro, F.; Bearpark, M.; Heyd, J. J.; Brothers, E.; Kudin, K. N.; Staroverov, V. N.; Kobayashi, R.; Normand, J.; Raghavachari, K.; Rendell, A.; Burant, J. C.; Iyengar, S. S.; Tomasi, J.; Cossi, M.; Rega, N.; Millam, J. M.; Klene, M.; Knox, J. E.; Cross, J. B.; Bakken, V.; Adamo, C.; Jaramillo, J.; Gomperts, R.; Stratmann, R. E.; Yazyev, O.; Austin, A. J.; Cammi, R.; Pomelli, C.; Ochterski, J. W.; Martin, R. L.; Morokuma, K.; Zakrzewski, V. G.; Voth, G. A.; Salvador, P.; Dannenberg, J. J.; Dapprich, S.; Daniels, A. D.; Farkas, Ö.; Foresman, J. B.; Ortiz, J. V.; Cioslowski, J.; Fox, D. J. Gaussian, Inc., Wallingford CT, 2009

7. NMR spectra of new compounds

MY13_19_1

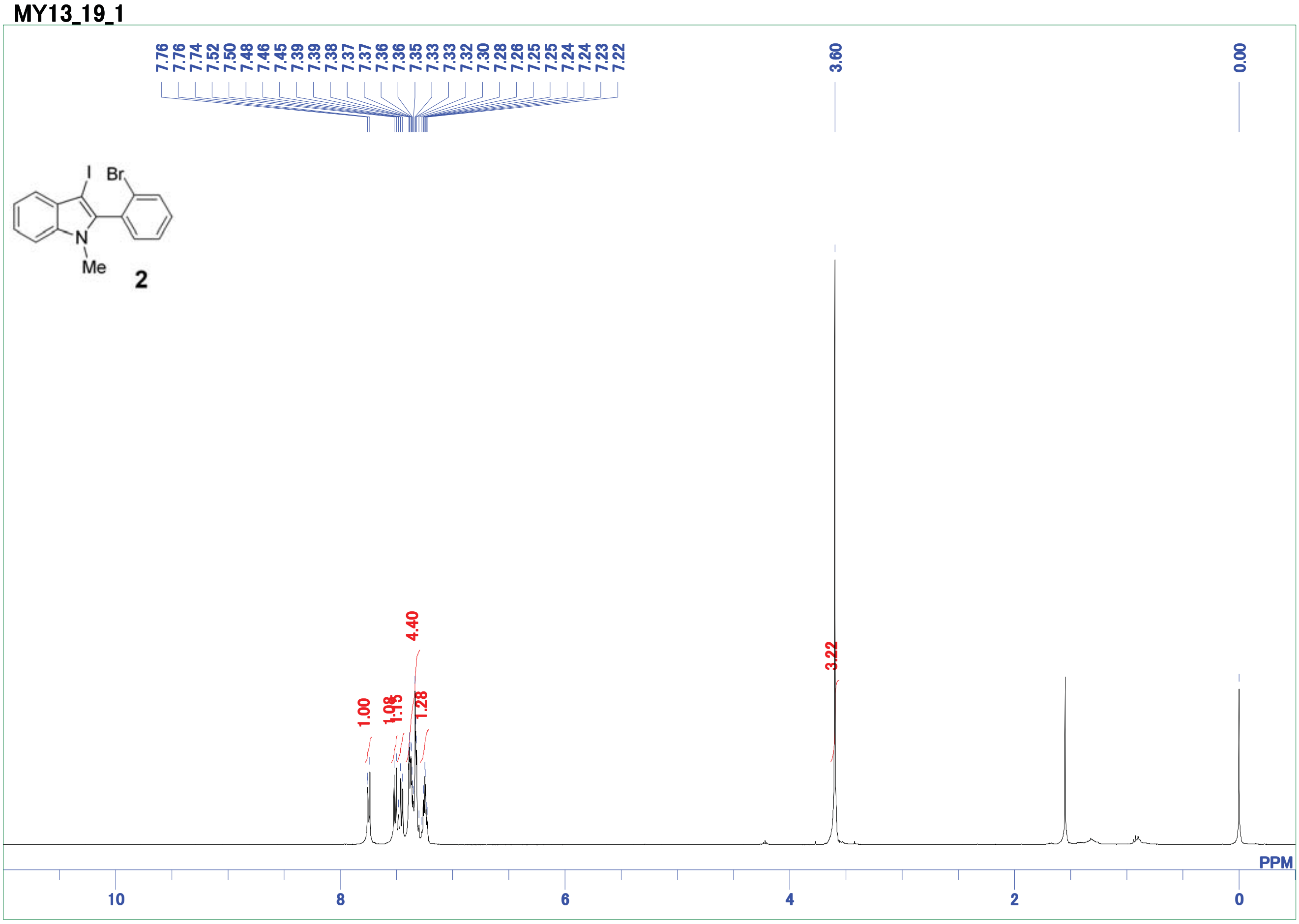

MY13-19-13C

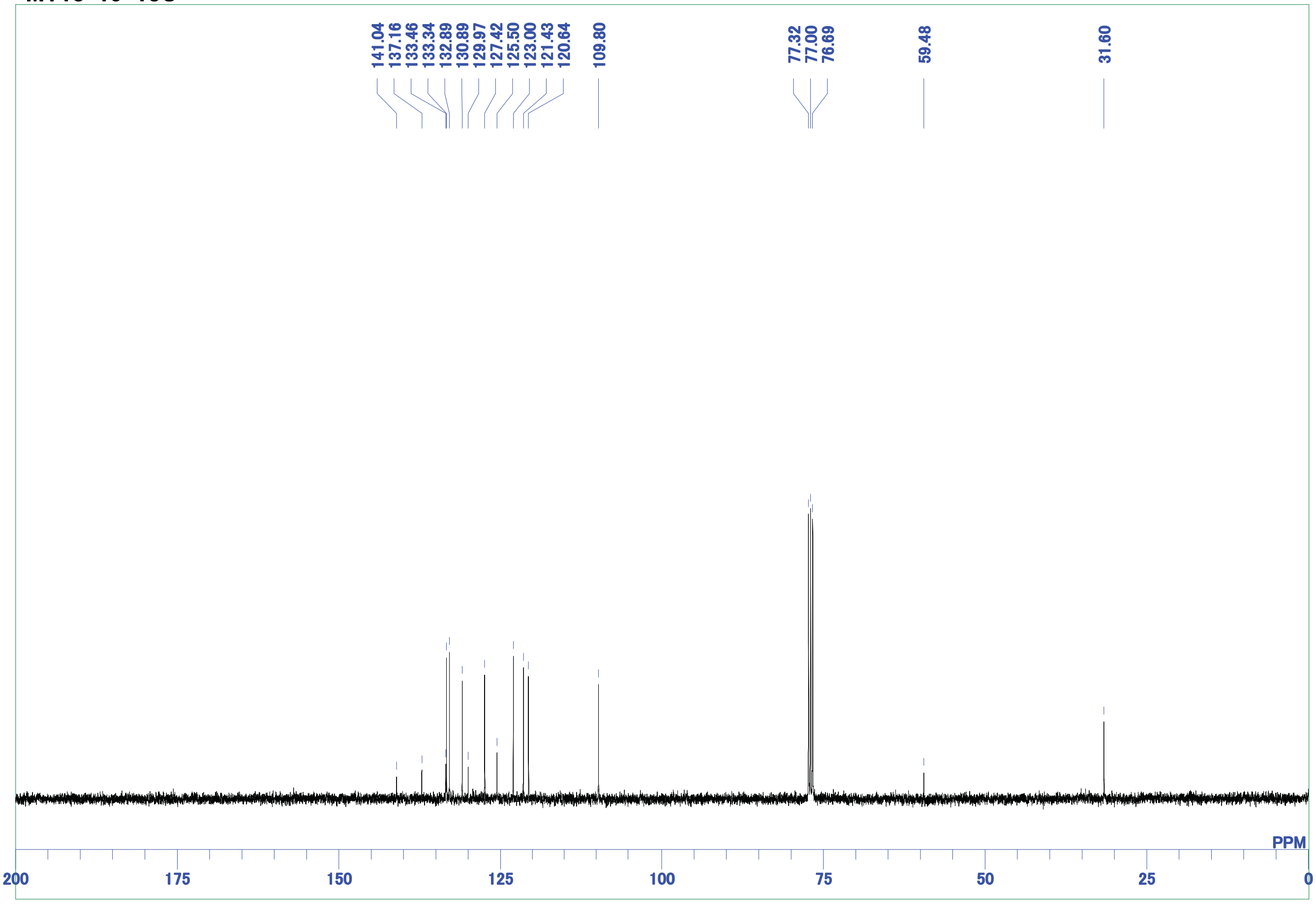

MY13_22_1

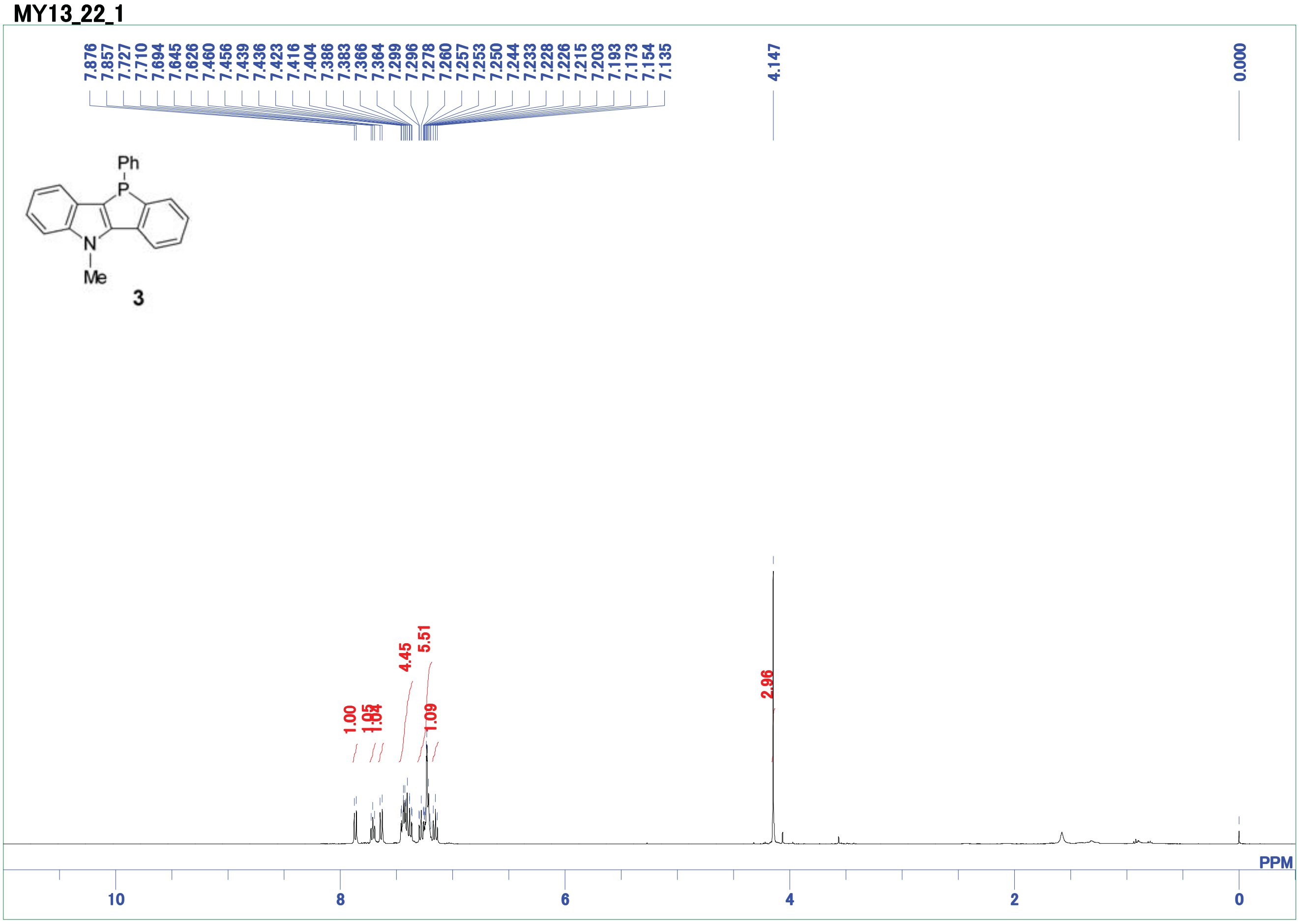

MY13_22_1_13C

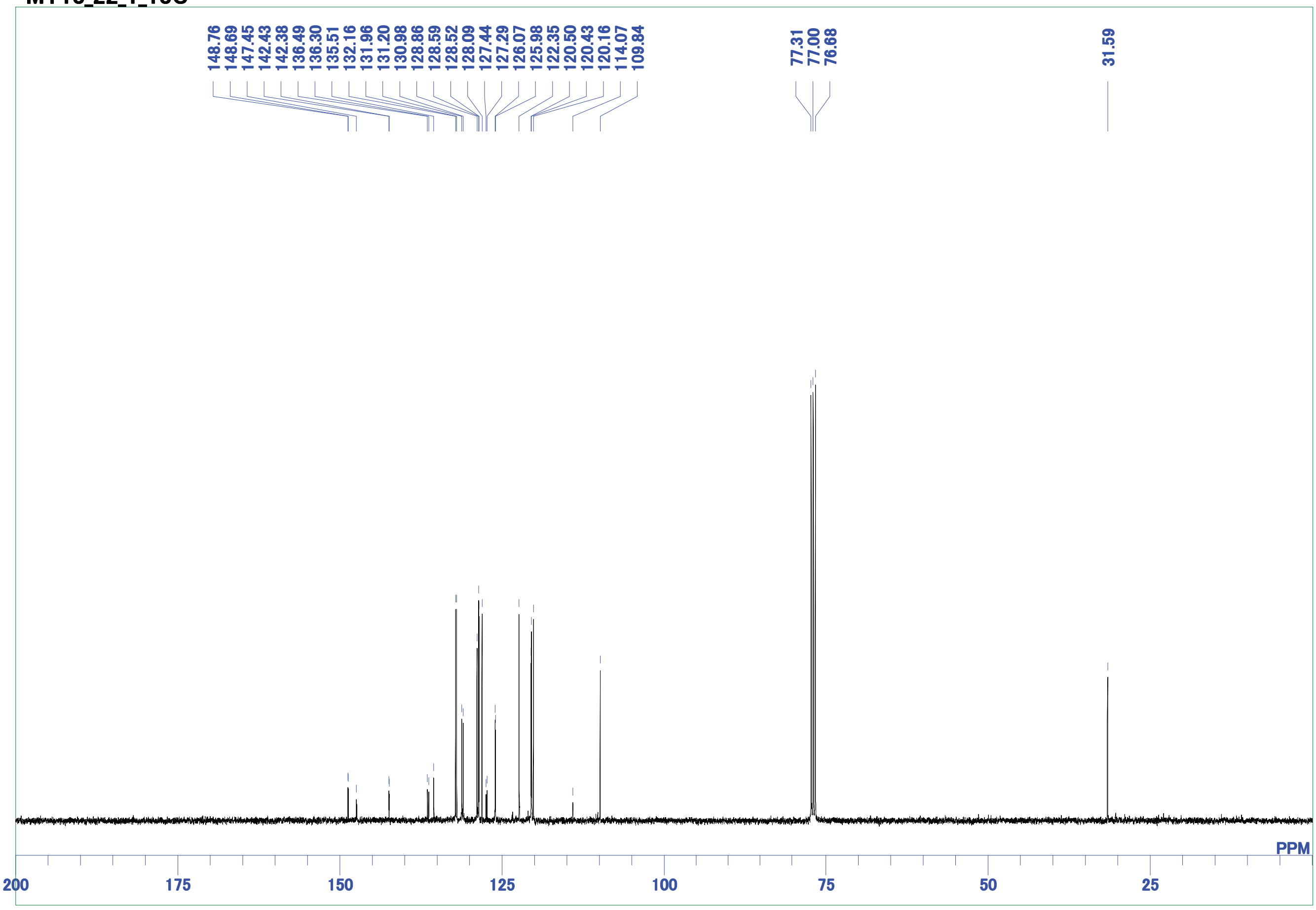

MY13_32_1

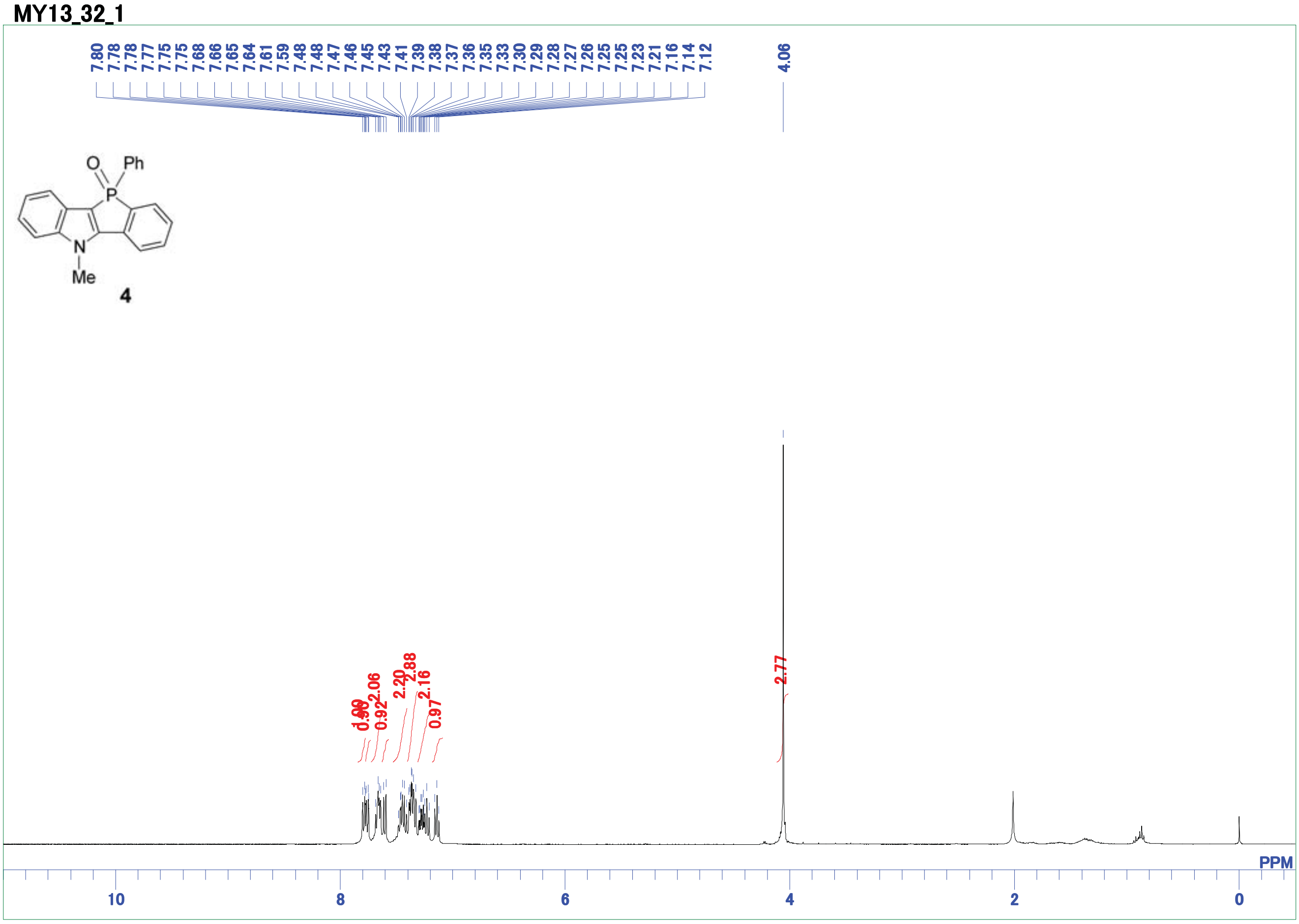

MY13_32_1_13C

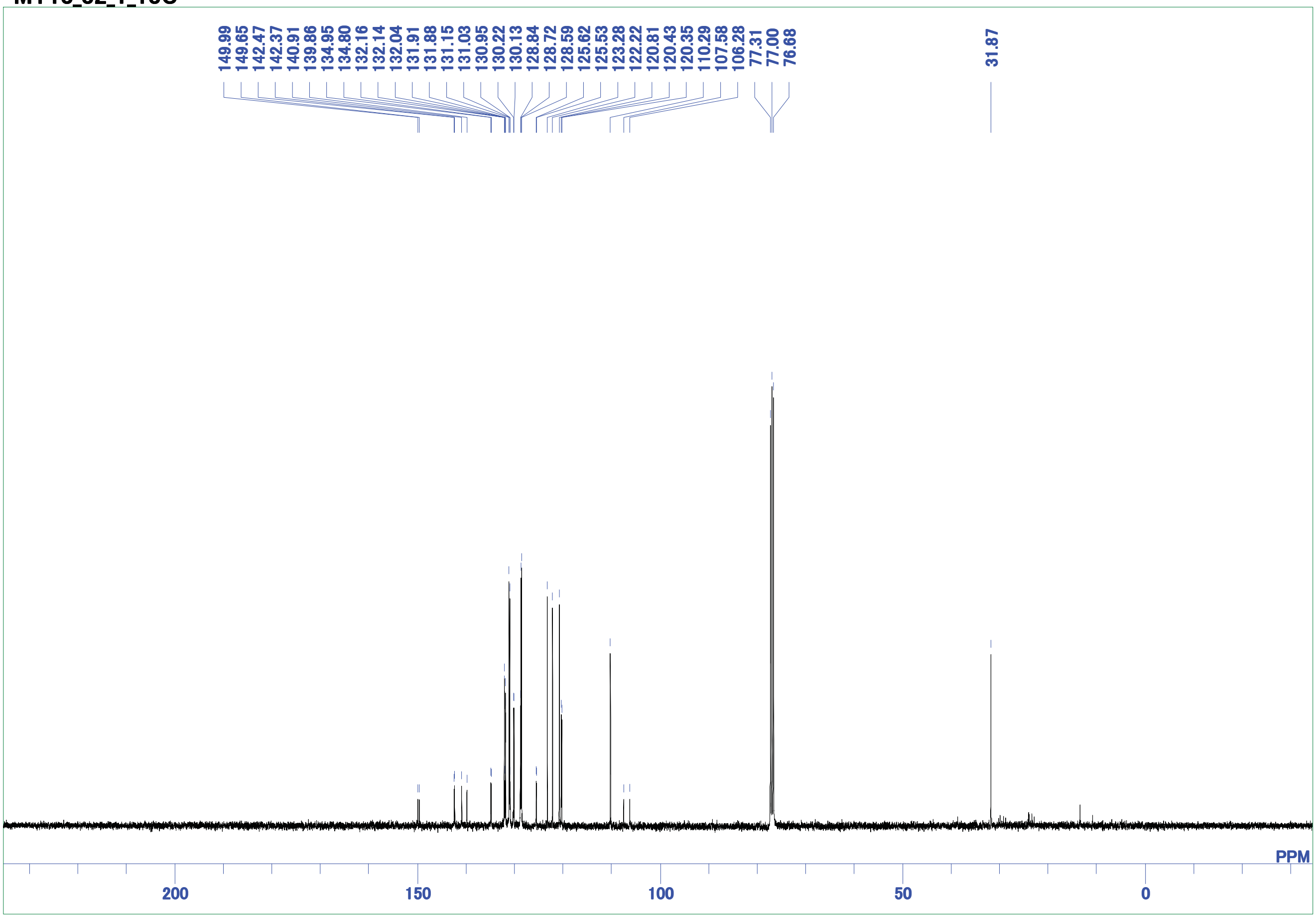

MM16_73_re

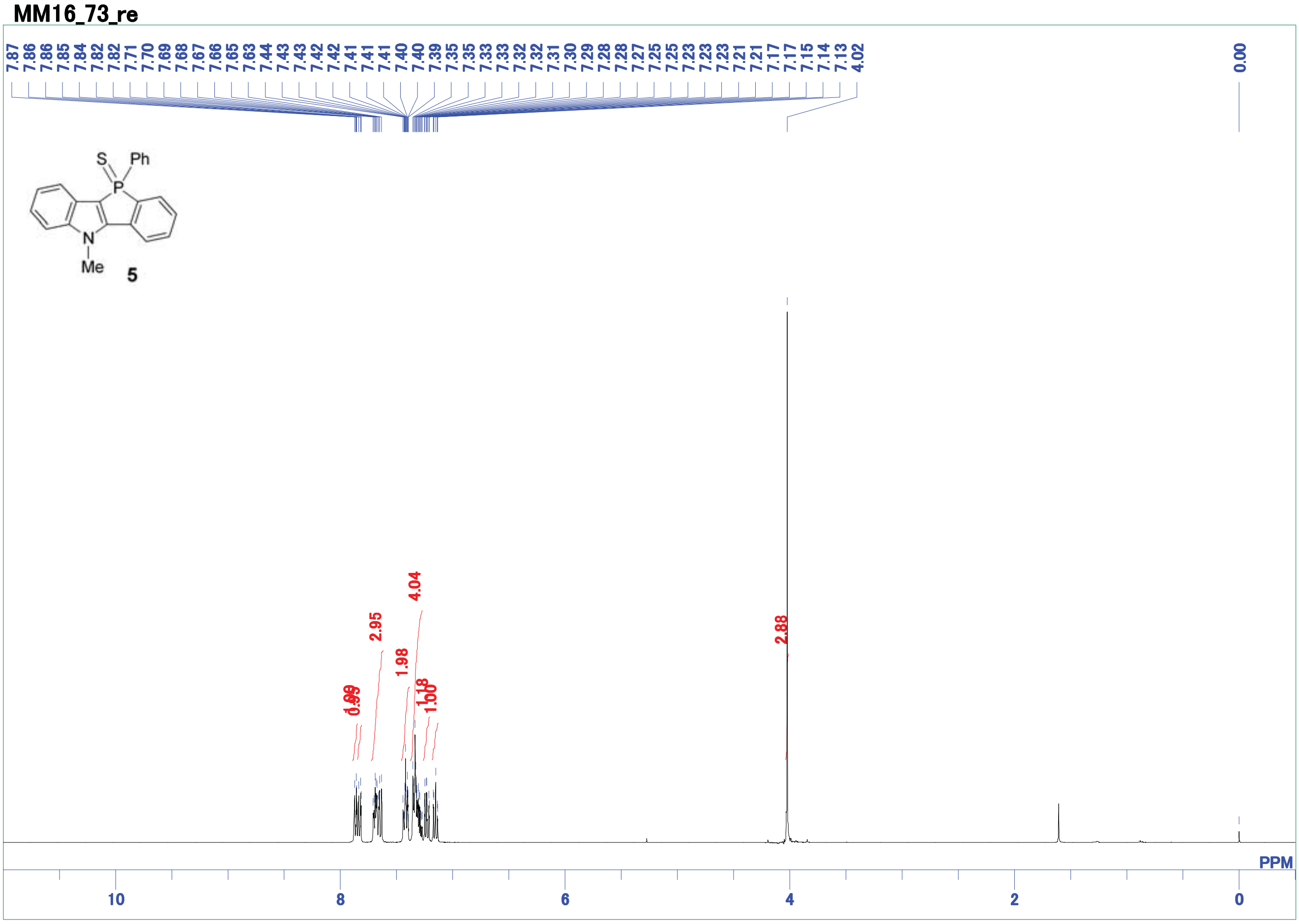

MM16_73_13C

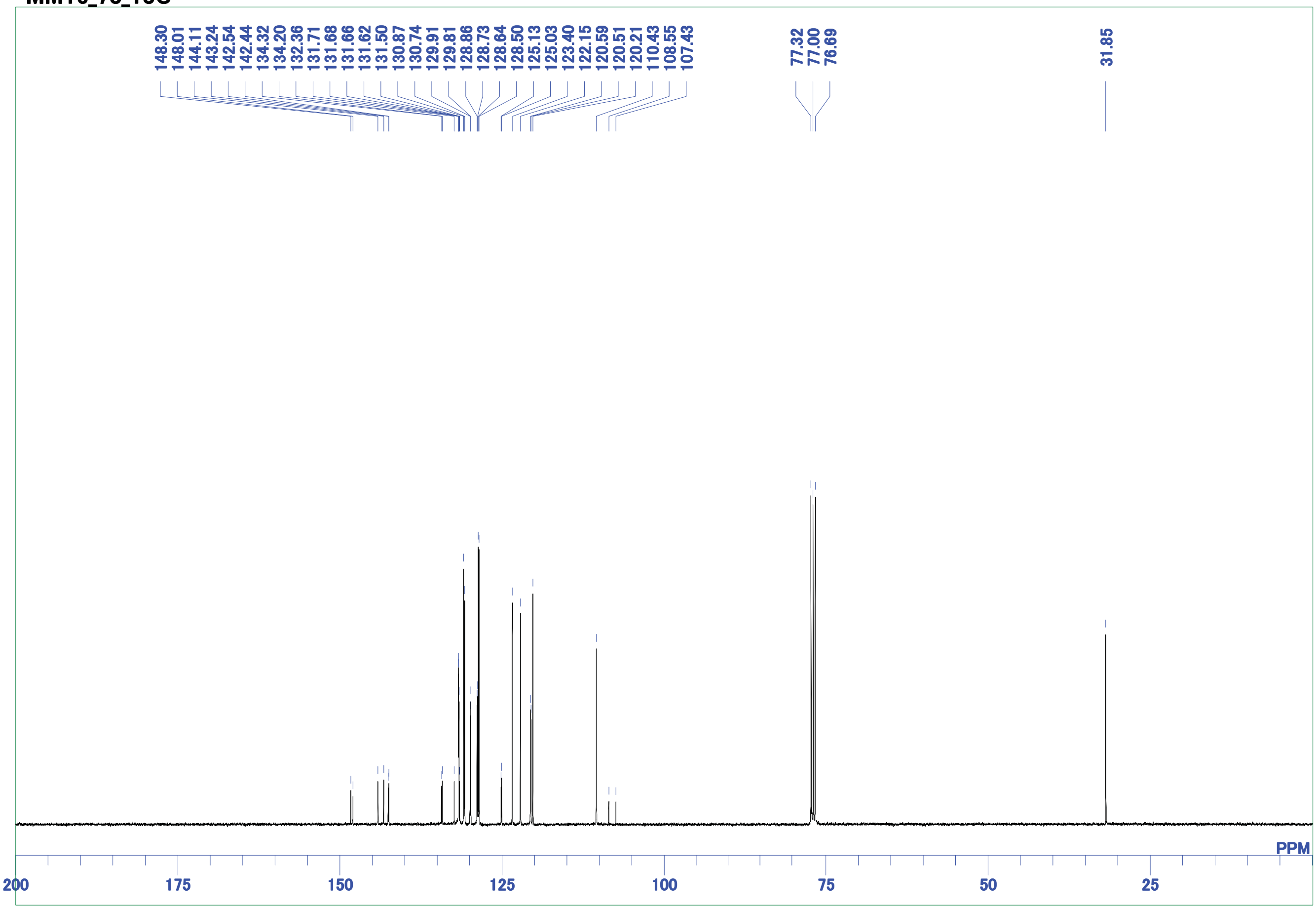

MM16_74_re

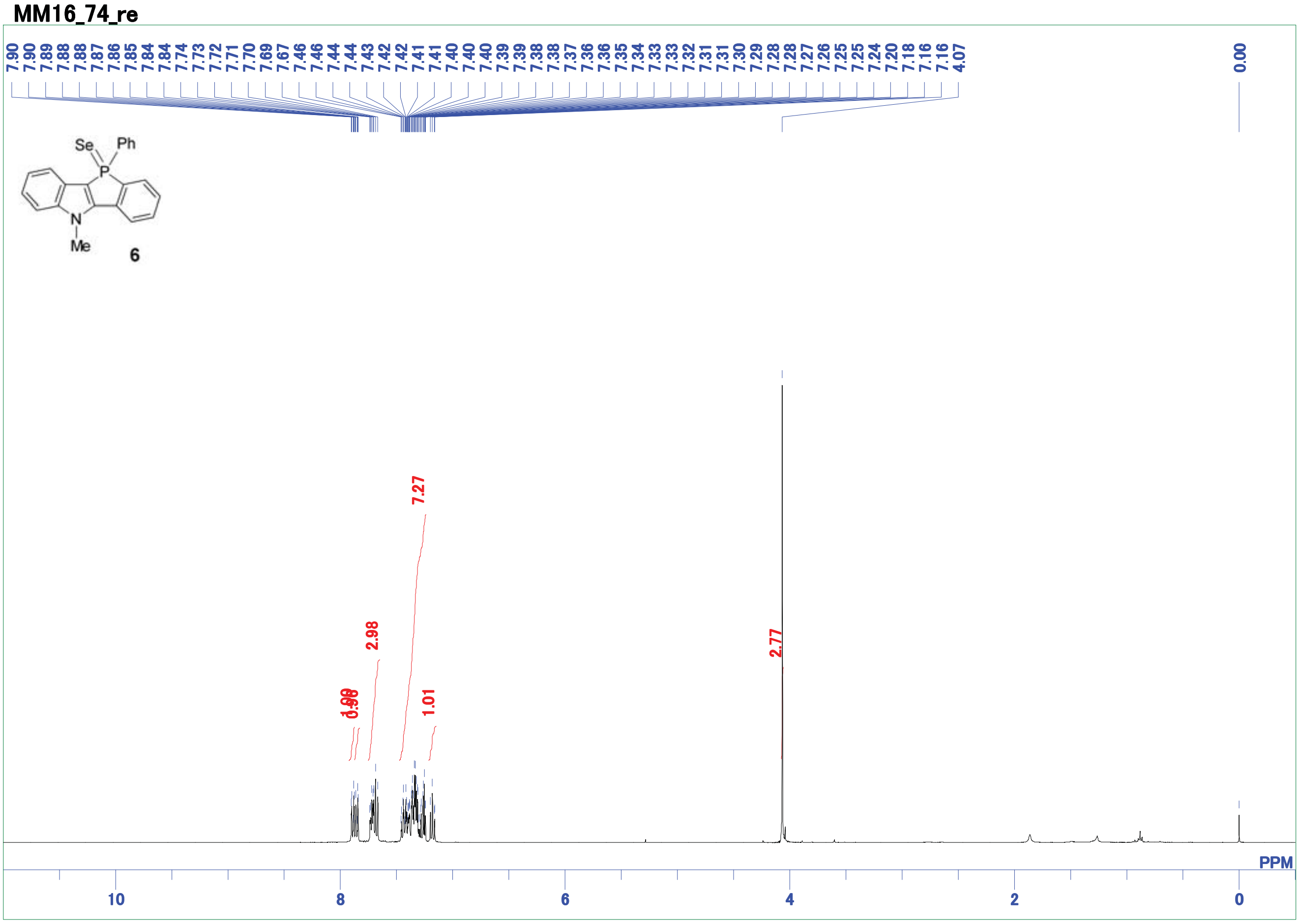

MM16_74_13C

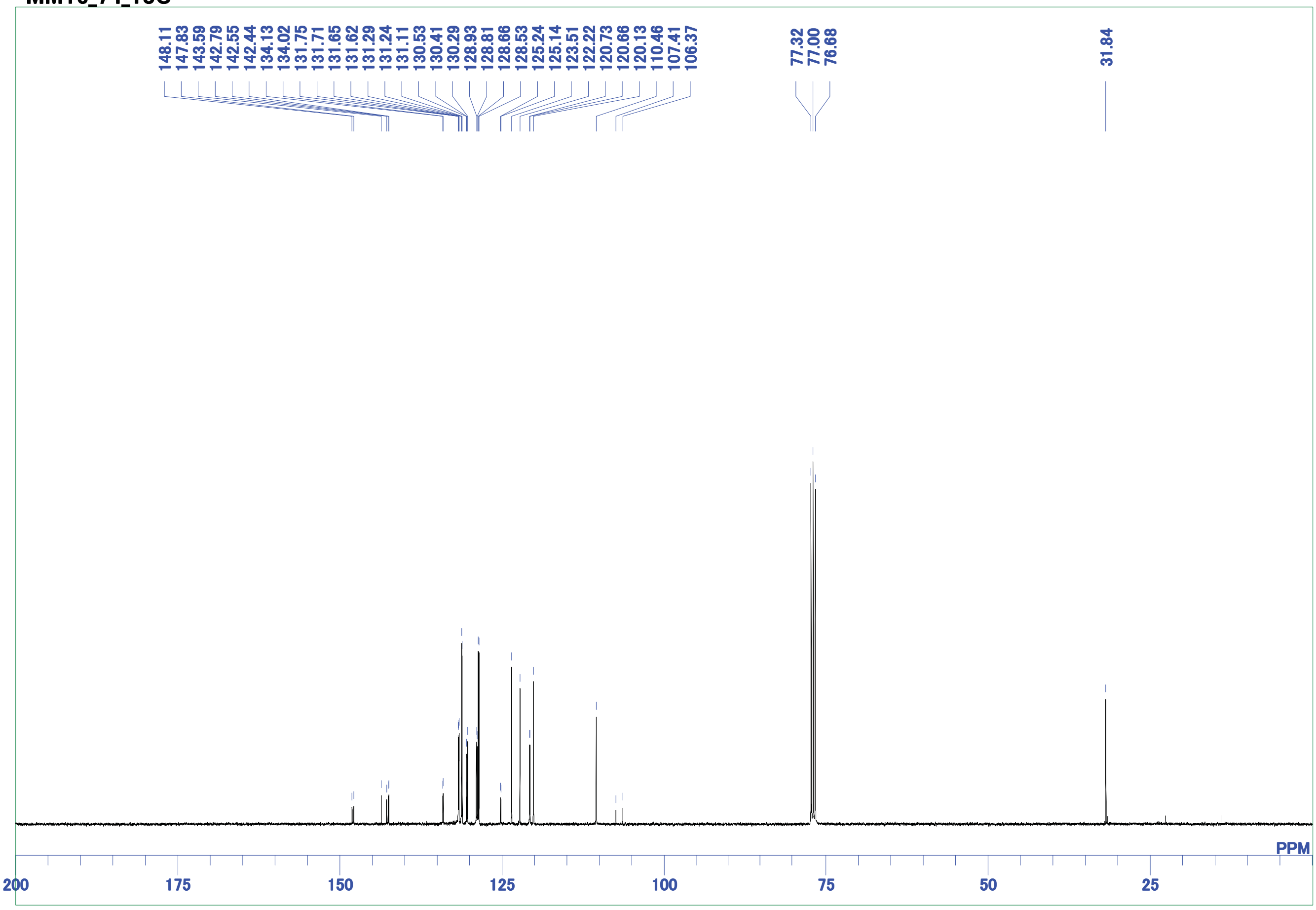

MM16_78_2nd

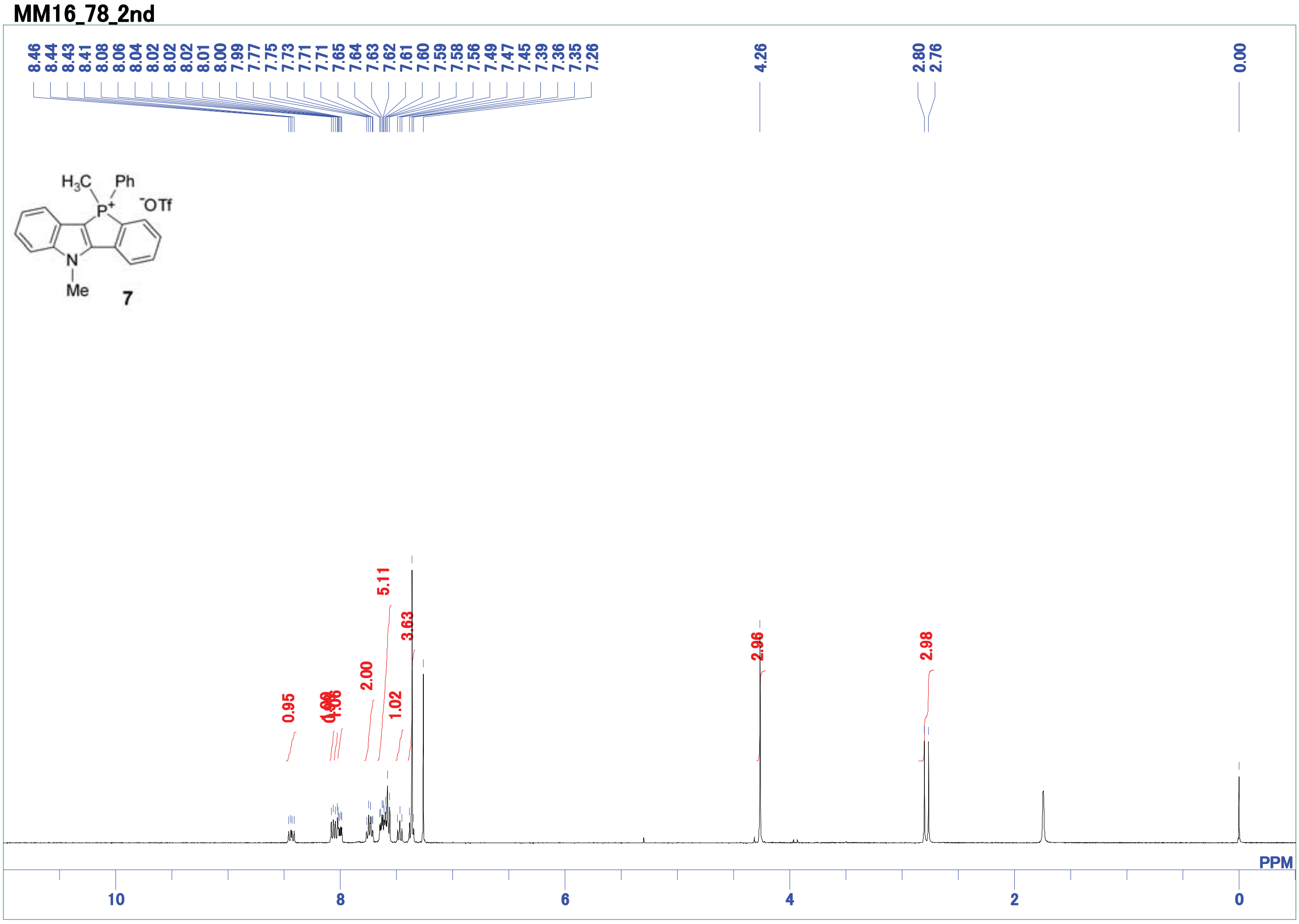

MM16_78_13C

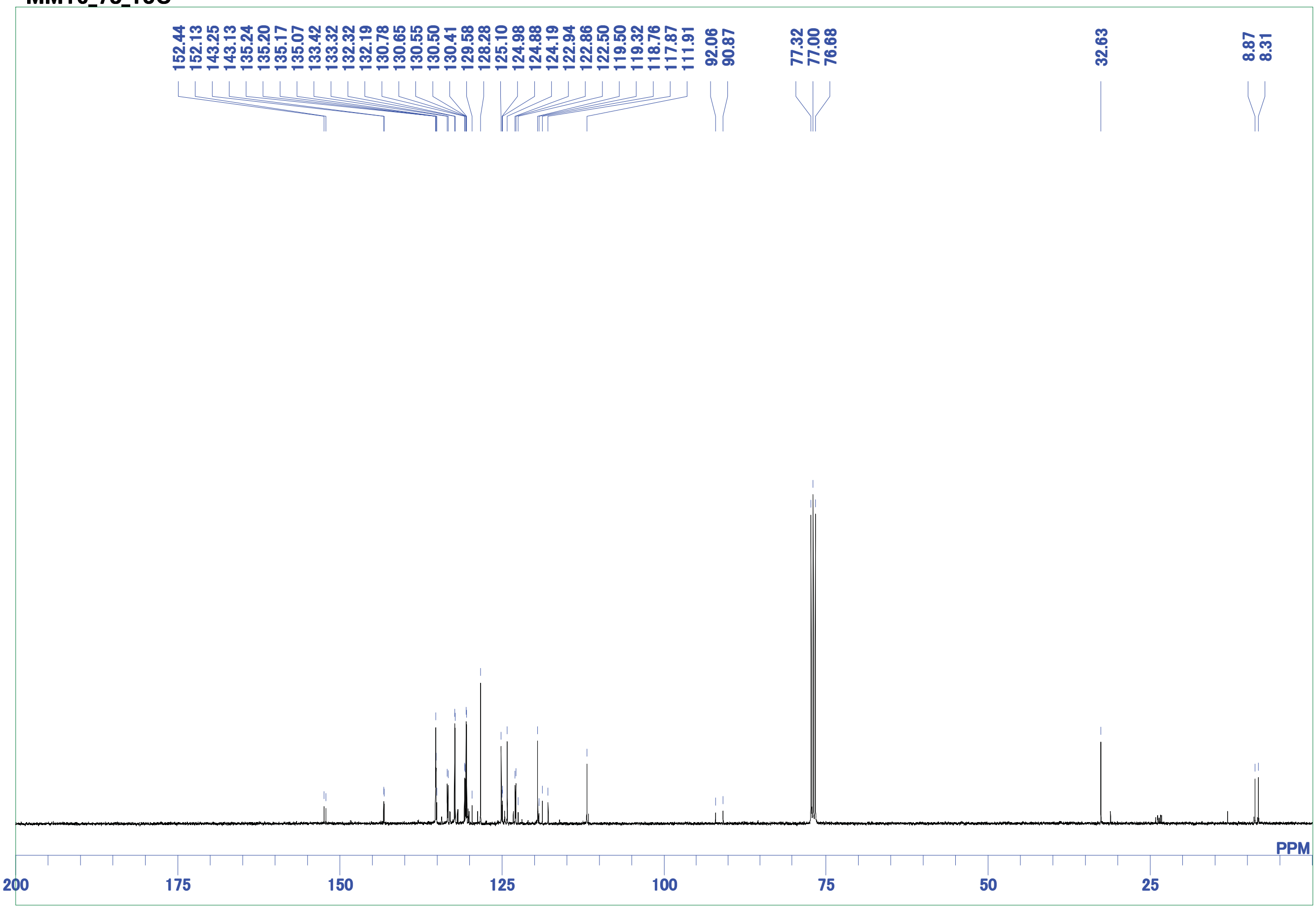

MM16_81_3rd

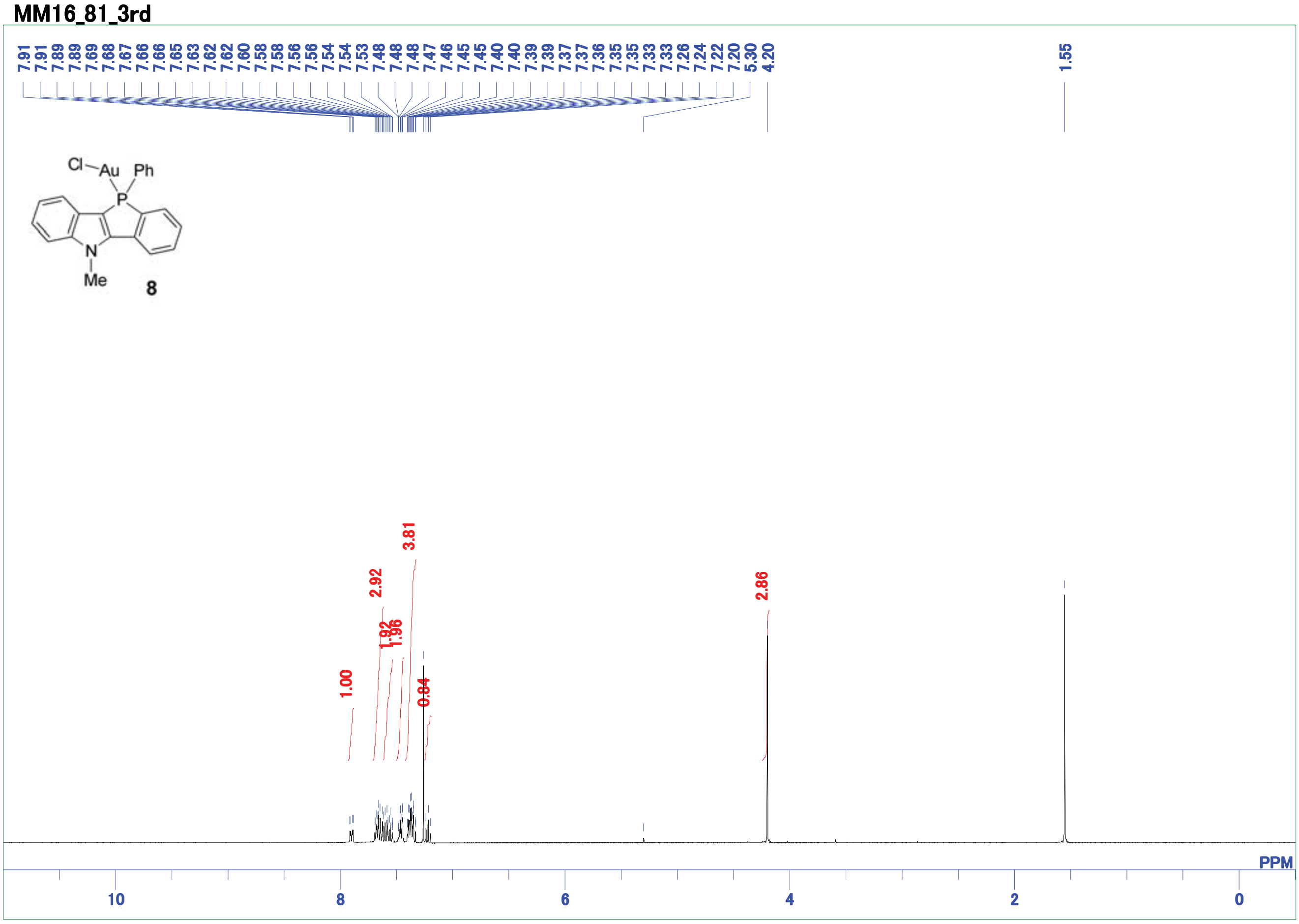

MM16_81_13C

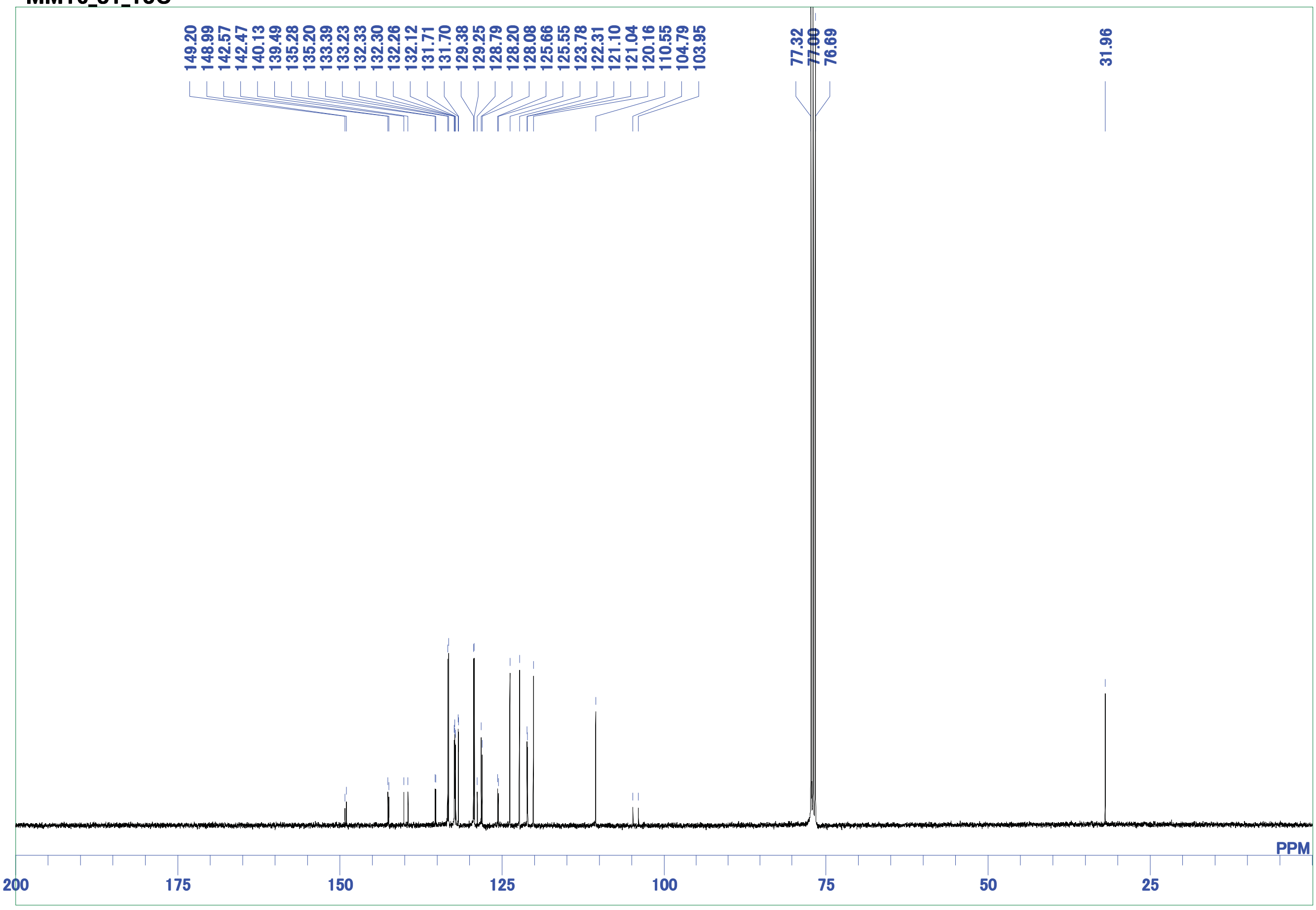

MM16_85_1st

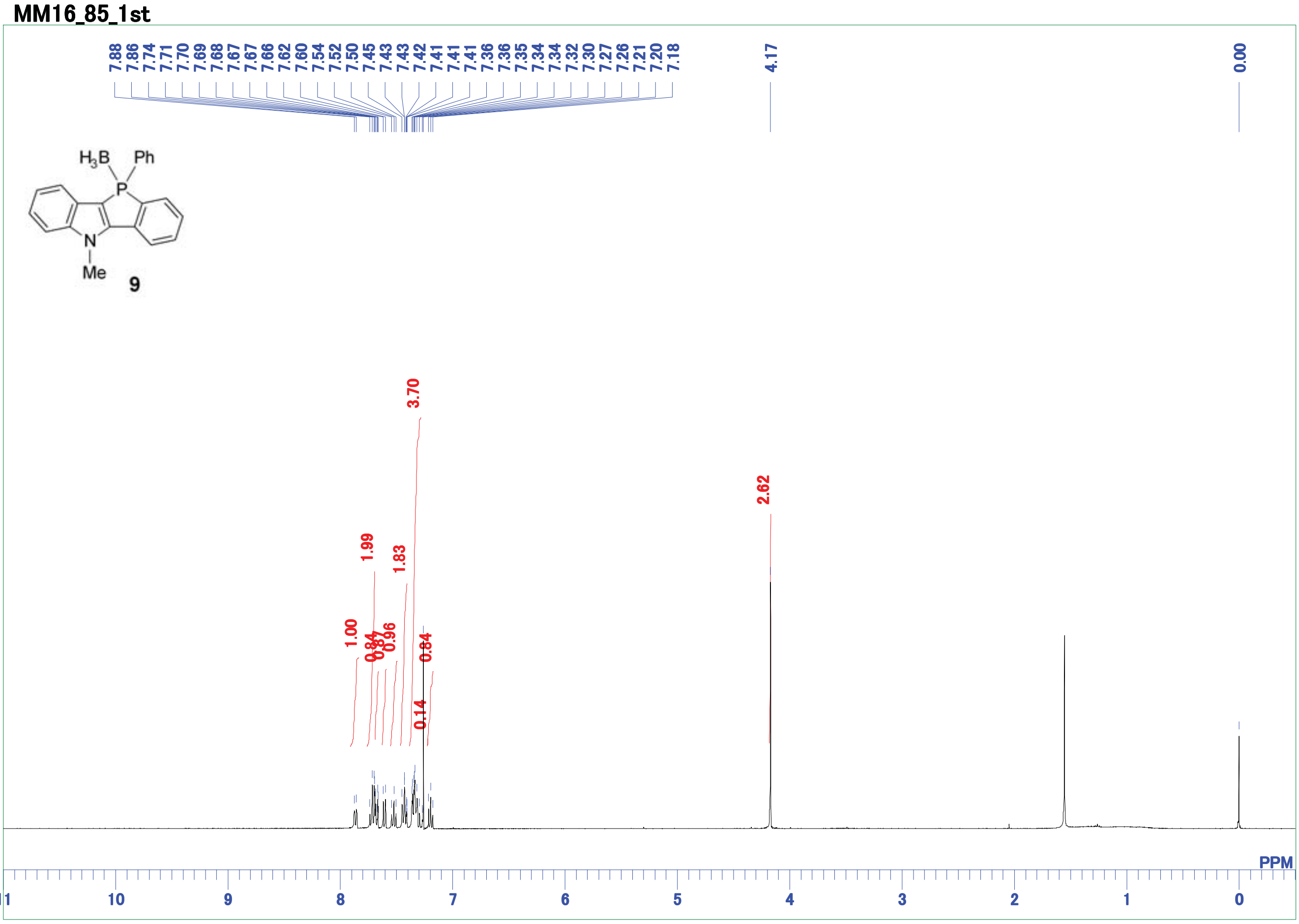

MM16_85_13C

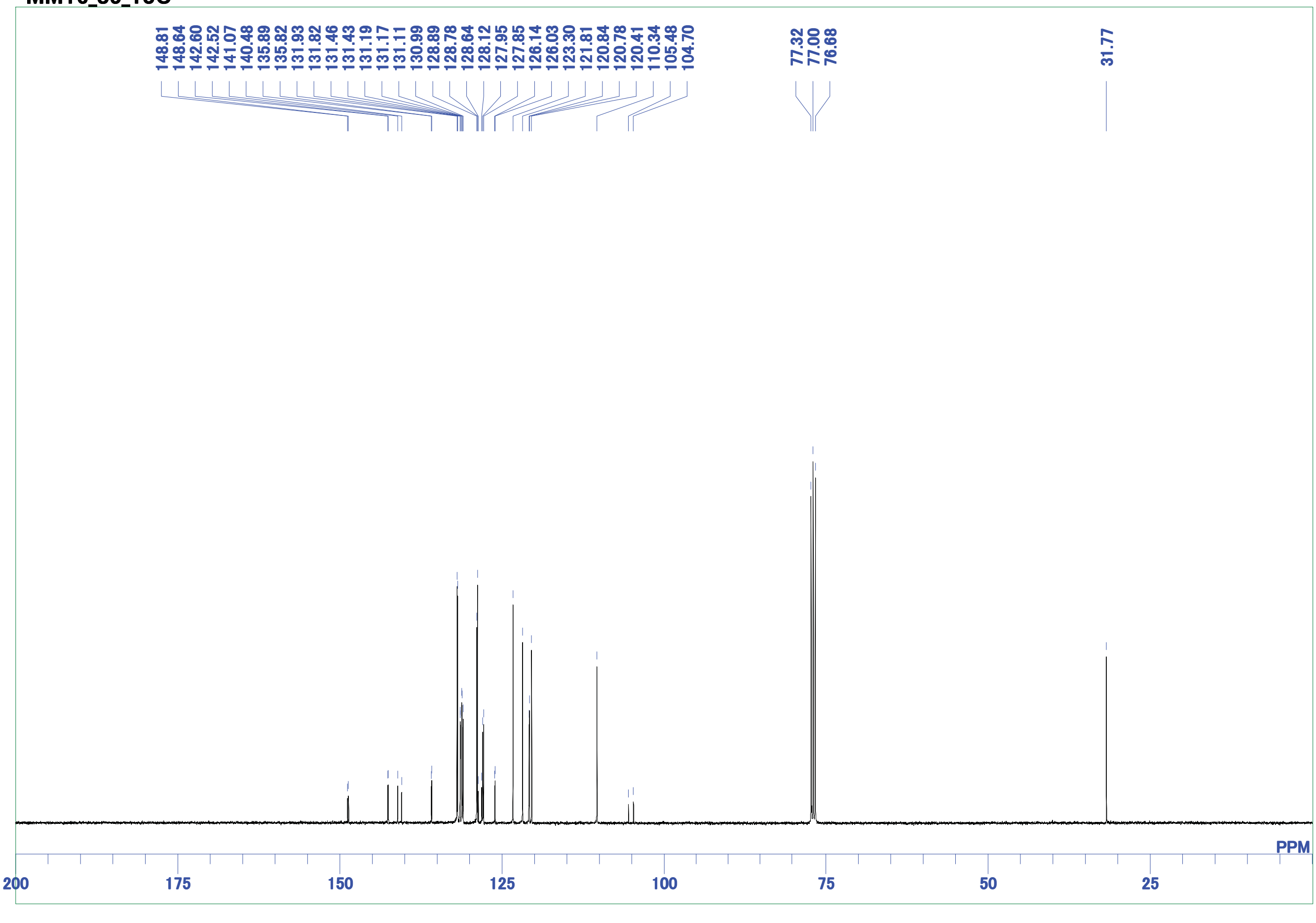

Supplement: File 1 — Experimental details, characterization data, and NMR spectra of all new compounds. [file Beilstein_J_Org_Chem-13-2304-s001.pdf]
